# Evolutionary history of LTR-retrotransposons among 20 Drosophila species

**DOI:** 10.1186/s13100-017-0090-3

**Published:** 2017-04-27

**Authors:** Nicolas Bargues, Emmanuelle Lerat

**Affiliations:** CNRS, UMR 5558, Laboratoire Biométrie et Biologie Evolutive, Université de Lyon, Université Claude Bernard Lyon 1, F-69622 Villeurbanne, France

**Keywords:** LTR-retrotransposons, Drosophila, Horizontal transfer, Transposable element dynamics

## Abstract

**Background:**

The presence of transposable elements (TEs) in genomes is known to explain in part the variations of genome sizes among eukaryotes. Even among closely related species, the variation of TE amount may be striking, as for example between the two sibling species, *Drosophila melanogaster* and *D. simulans*. However, not much is known concerning the TE content and dynamics among other Drosophila species. The sequencing of several Drosophila genomes, covering the two subgenus *Sophophora* and *Drosophila,* revealed a large variation of the repeat content among these species but no much information is known concerning their precise TE content. The identification of some consensus sequences of TEs from the various sequenced *Drosophila* species allowed to get an idea concerning their variety in term of diversity of superfamilies but the used classification remains very elusive and ambiguous.

**Results:**

We choose to focus on LTR-retrotransposons because they represent the most widely represented class of TEs in the *Drosophila* genomes. In this work, we describe for the first time the phylogenetic relationship of each LTR-retrotransposon family described in 20 Drosophila species, compute their proportion in their respective genomes and identify several new cases of horizontal transfers.

**Conclusion:**

All these results allow us to have a clearer view on the evolutionary history of LTR retrotransposons among *Drosophila* that seems to be mainly driven by vertical transmissions although the implications of horizontal transfers, losses and intra-specific diversification are clearly also at play.

**Electronic supplementary material:**

The online version of this article (doi:10.1186/s13100-017-0090-3) contains supplementary material, which is available to authorized users.

## Background

It is now clearly established that the presence of transposable elements (TEs), which can make up a large and variable proportion of eukaryotic genomes, explains in part the variations of genome sizes [1–3]. Even among closely related species, the variation of TE amount may be striking, as it is the case for the two sibling species, *Drosophila melanogaster* and *D. simulans*. Indeed, it has been shown since a long time that *D. melanogaster* harbors around three times more TEs than *D. simulans* although both species share a lot of similarities like a similar worldwide geographical distribution or the fact that they are almost phenotypically identical [4, 5]. In a previous study, we have analyzed 12 LTR retrotransposons and three non-LTR retrotransposons described in detail in *D. melanogaster* and known to present variations in copy number among natural populations of *D. simulans* [6]. We determined their copy numbers and structures in the related species of the *melanogaster* subgroup *D. simulans*, *D. sechellia*, and *D. yakuba.* Our results showed that *D. melanogaster* appears like a special case among these other drosophila species with a lot of full-length and potentially active copies whereas more ancient and degraded sequences were present in the three other species. This was pointing out the fact that relying only on one genome from one given species is not enough to fully understand the dynamics of TEs in related species.

Not much is known concerning the TE content and dynamics among Drosophila species expected in *D. melanogaster*. The sequencing of 11 other Drosophila genomes, covering the two subgenus *Sophophora* and *Drosophila,* revealed a large variation of the euchromatic repeat content among these species, going from ~2.7% in *D. simulans* and *D. grimshawi* to ~25% in *D. ananassae* [7]. Some years latter, the sequencing of eight additional species from the same subgenus from the consortium modENCODE (https://www.hgsc.bcm.edu/arthropods/drosophila-modencode-project) was performed but no much information is known concerning the TE content in these last species. The only studies that made the effort to decipher TE dynamics in Drosophila species other than the model species *D. melanogaster* were either centered on particular species like for example on *D. buzzatii* and *D. mojavensis* [8] or on a particular type of TEs like the exploration of the dynamics of mariner DNA transposons [9], Roo and RooA LTR retrotransposons [10] or of DINE-1 elements [11]. The presence of consensus sequences in the Repbase database [12] of TEs from the various sequenced *Drosophila* species is certainly helping to get an idea concerning their variety in term of diversity of families inside these species. However, the classification remains particularly elusive and ambiguous. For example, from the name and annotation, it is not possible to tell the difference between the LTR-retrotransposons BEL1 and BEL-1 from *D. virilis* nor it is possible to consider that the element Gypsy-1 present in *D. rhopaloa* is homologous to the element Gypsy-1 in *D. ficusphila*.

A clearer view on TE evolutionary relationship among *Drosophila* species is thus needed to understand how TEs can be maintained in genomes and what mechanisms make them diversify inside a genome. This is what we have intended to perform in this work and to do so, we choose to focus on LTR-retrotransposons because they are known to usually represent the most widely represented class of TEs in the *Drosophila* genomes for which we have the sequences [7, 13] and because they can be subject to numerous horizontal transfers in these species [6, 14–18]. Moreover, our previous work has confirmed the existence of sequence variants, especially in *D. simulans*, that could have emerged from recombination between closely related families, giving a lead toward a mechanism of formation of new families that remains to be explored [6]. However, to be able to determine such events, it is indispensable to have a clear idea about the evolutionary links among the various families present in the *Drosophila* genomes.

LTR-retrotransposons are one of the main subclasses among the elements transposing by a “copy-and-paste” mechanism via an RNA-intermediate [19]. They possess Long Terminal Repeat (LTR) sequences at their extremities and usually present two open reading frames encoding for the proteins necessary for their transposition, especially the *gag* and *pol* genes. According to the protein domain order found in the *pol* gene, three superfamilies have been described: *Ty1/Copia*, *Ty3/Gypsy*, and *BEL/Pao* [20]. In this work, we have defined new reference sequences corresponding to consensus of families never described until now in the species from the *melanogaster* subgroup using a *de novo* approach and we used, in addition, described reference elements to 1) determine accurately their phylogenetic positions inside each superfamily; 2) detect horizontal transfers events, especially among the *melanogaster* subgroup and identify potential losses and intra-specific diversification events; 3) compute their proportion in their respective genomes. Our results allowed us to determine for each family from the 20 Drosophila species to which exact group inside the superfamilies they belong. Although vertical transmission, along with losses and intra-specific diversity, seem to be the most common scenario to explain the phylogenetic pictures we observed, we also identified some new cases of HTs especially among certain species from the *melanogaster* subgroup and detected some new groups of TEs that are absent from *D. melanogaster*, *D. simulans*, *D. sechellia*, and *D. erecta*. All these results allow us to have a clearer view on the evolutionary history of LTR retrotransposons among these 20 *Drosophila* species.

## Results and discussion

### Identification of new reference elements in the species from the *melanogaster* subgroup

For the four drosophila species from the *melanogaster* subgroup, we obtained 1501 candidates for *D. yakuba*, 603 for *D. simulans*, 1681 for *D. sechellia*, and 766 for *D. erecta* when using the LTRharvest program on their genome assemblies. We then retained the sequences corresponding to real LTR-retrotransposons, the remaining being false positives. The proportion of these false positives was quite high (between 77 and 85%) compared to what was expected by the use of LTRharvest on the *D. melanogaster* genome [21, 22]. This could be due to the fact that in *D. melanogaster*, the TEs correspond to mainly full-length elements whereas in the other species full-length elements are more rare, as it has been observed when analyzing 12 LTR-retrotransposons [6].

We thus retained 217 sequences in *D. yakuba*, 103 in *D. simulans*, 325 in *D. sechellia*, and 178 in *D. erecta*. For each species, we clustered the sequences in group of families, according to the 80-80-80 rule, for which we constructed a consensus. We thus obtained 54 different consensus (or references) for *D. yakuba*, 46 for *D. simulans*, 59 for *D. sechellia*, and 22 for *D. erecta*. To determine if some of them were already described families we compared them to the consensus present in the Repbase database as well as sequences present in Flybase and Genbank, or described only in the literature [18]. We considered a consensus to be already described if we were able to find a match with more than 98% nucleotidic identity with a described element in the same species. In the case of *D. yakuba*, 25 consensus appeared to be new families, 43 in *D. simulans*, 57 in *D. sechellia*, and 21 in *D. erecta*. These results indicate that a lot of the identified references correspond to new elements from known families in *D. melanogaster* but never described for in these four species to date. This implies that a still large amount of unknown references need to be discovered and that the databases, and especially Repbase, are not exhaustive for these particular species. Interestingly, a large proportion of them (17 in *D. yakuba*, 25 in *D. simulans*, 32 in *D. sechellia*, and four in *D. erecta*) displayed a very high percentage identity (over 95% on average) with elements from *D. melanogaster*, giving some hints toward potential HTs that need to be explored, since the global % identity is about 93.6% between *D. melanogaster* and both *D. simulans* or *D. sechellia*, and about 68% between *D. melanogaster* and both *D. yakuba* and *D. erecta*.

In total, we thus have identified 141 new reference elements (or families) from these four *Drosophila* species over a total of 206 elements (see Additional file 1 for their fasta sequences), with 76 of them corresponding to elements almost identical to references from *D. melanogaster* (*i. e.* with a mean % identity over 98% when comparing the entire nucleotidic sequences). Additionally, we found two new elements from the *Ty1/Copia* superfamily in *D. melanogaster* corresponding to COPIA2bis and new_Xanthias, although this genome is particularly well studied and annotated.

### Phylogenetic analyses of the three main superfamilies of LTR retrotransposons reveal a dynamics mainly constituted by vertical transmissions, several cases of horizontal transfers but also internal species diversification

We have built reference sequences, representative of a given family, corresponding to consensus obtained from the alignment of copies detected using a *de novo* approach in the four species of the *melanogaster* subgroup. However, this approach did not allow us to retrieve the sequences of all the known LTR retrotransposon families in some species, probably due to the lack of full-length copies for these missing elements. Indeed, a drawback of a *de novo* approach like *LTRharvest* is that it is only able to identify full-length or nearly full-length elements, with two conserved LTRs at each extremity. We thus added the missing families whose sequences were present in Repbase, corresponding to 35 sequences in *D. yakuba*, 15 sequences in *D. simulans*, 10 sequences in *D. sechellia*, and one sequence in *D. erecta* (see Additional file 2: Table S1 and Table 1 for the number of reference sequences in each superfamily for each species).

**Table 1:** Number of reference sequences from each superfamily

Species	*Ty1/Copia*	*BEL/Pao*	*Ty3/Gypsy*	Total
*D. ananassae*	8	22	44	74
*D. biarmipes*	1	4	14	19
*D. bipectinata*	3	12	26	41
*D. elegans*	2	18	44	64
*D. erecta*	0	2	20	22
*D. eugracilis*	3	5	14	22
*D. ficusphila*	1	5	13	19
*D. grimshawi*	4	3	8	15
*D. kikkawai*	1	1	5	7
*D. melanogaster*	6	7	48	61
*D. mojavensis*	4	9	10	23
*D. persimilis*	3	14	16	33
*D. pseudoobscura*	2	7	26	35
*D. rhopaloa*	0	7	17	24
*D. sechellia*	6	9	51	66
*D. simulans*	6	8	44	58
*D. takahashi*	2	22	20	44
*D. virilis*	1	6	18	25
*D. willistoni*	9	21	61	91
*D. yakuba*	4	12	44	60

For each of the three main superfamilies, *Ty1/Copia, BEL/Pao* and *Ty3/Gypsy*, we then reconstructed the phylogeny of the various families based on the pol protein, which contains the most conserved enzymatic domains in the LTR-retrotransposons [19, 20].

#### The phylogenetic analysis of *Ty1/Copia* families reveals a history made of a majority of vertical transmissions, with many losses

**Fig. 1:**
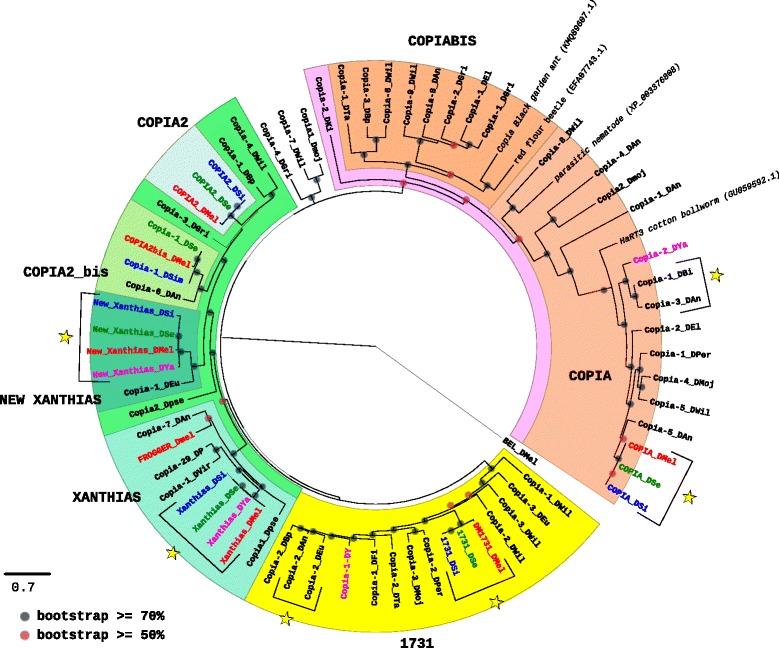
Maximum likelihood phylogenetic tree based on the polyprotein amino acid sequences of *Ty1/Copia* elements. Only bootstrap values greater than 50% (*red dots*) or greater than 70% (*black dot*) are indicated. The tree has been rooted by the BEL element from *D. melanogaster*. The names of the species are abbreviated as follows: DAn, *D. ananassae*; DBi, *D. biarmipes*; DBp, *D. bipectina*; DEl, *D. elegans;* DEu*, D. eugracilis;* DFi, *D. ficusphila*; DGri, *D. grimshawi;* DKi, *D. kikkawai*; DMel, *D. melanogaster* (*in red*); DMoj/Dmoj; *D. mojavensis*; DPer/DP, *D. persimilis*; Dpse, *D. pseudoobscura*; DSe, *D. sechellia* (*in green*); DSi, *D. simulans* (*in blue*); DTa, *D. takahashi*; DVir, *D. virilis*; DWil, *D. willistoni*; Dya/DY, *D. yakuba* (*in pink*). Four sequences from other organisms are included. *Yellow stars* represent cases of confirmed horizontal transfers (see details in Additional file 3: Figure S1a)

Families from the *Ty1/Copia* are not the most diversified in the *drosophila* species (Table 1). However, *D. ananassae* and *D. willistoni* present the highest number of different references with respectively eight and nine families, whereas no reference has been identified for *D. erecta* and *D. rhopoloa*. The phylogenetic tree based on the pol protein is represented on Fig. 1. Three major clades, highlighted in pink, green, and yellow, well supported by the bootstrap values, can be separated in several subclades. Some of them correspond to the classical known groups, which are COPIA (in light orange), 1731 (in yellow), and XANTHIAS (in blue green) [20]. We also were able to identify some new subclades that we named COPIABIS in orange, due to its proximity to COPIA, COPIA2 in light blue, COPIA2BIS in light green, and NEW XANTHIAS in dark green. The COPIABIS subclade has the particularity to present no sequence from the *melanogaster* subgroup species. The species harboring families from this subclade are restricted to *D. ananassae*, *D. elegans*, *D. bipectinata*, *D. takahashi*, *D. willistoni*, and *D. grimshawi*. This patchy distribution among species from both Drosophila and Sophophora subgenus could indicate that elements from this subclade have been lost in the other species.

An interesting point concerning the families present in the species of the *melanogaster* subgroup is that their nucleotidic sequences are particularly similar, especially among the three species *D. melanogaster*, *D. simulans*, and *D. sechellia,* and sometimes *D. yakuba*. In order to determine if this could be due to HT events, we tested the hypothesis for the elements COPIA2, COPIA2bis, new_Xanthias, Xanthias, 1731 and COPIA. In the cases of new_Xanthias, Xanthias, 1731 and COPIA, the VHICA method [23] allowed us to confirm HT events between *D. melanogaster* and *D sechellia* (COPIA, 1731, Xanthias and new_Xanthias) and between *D. yakuba* and *D. simulans* (Xanthias and new_Xanthias) (Additional file 3: Figure S1a). Excepted new_Xanthias and Xanthias, these HTs were already documented in previous studies for the same species [14, 17, 23]. Two new cases of HTs were also detected implying species outside the *melanogaster* subgroup for the elements Copia-3_DAn from *D. ananassae* and Copia-1_DBi from *D. biarmipes* on one hand, and the elements Copia-2_DAn from *D. ananassae*, and Copia-2_Deu from *D. eugracilis* on the other hand (Additional file 3: Figure S1a). We however did not observe any HT implying one of the numerous families from *D. willistoni* and the Copia element of *D. melanogaster*, contrary to what was proposed [24]. To make sure that it could not be due to a missing reference in Repbase for *D. willistoni,* we performed a blastn search in the genome sequence of *D. willistoni* using the Copia element from *D. melanogaster*. However, we did not find any significant matches corresponding to a nearly identical sequence as found by PCR approaches [24]. Such a situation is not that unusual. Indeed, the genome sequence of *D. melanogaster* is empty of the horizontally transferred DNA transposon P that was introduced from *D. willistoni* some decades ago [25] simply because the strain that has been sequenced is an old lab strain taken in nature before the HT happened [26]. It is thus possible that the sequenced genome of *D. willistoni* is not harboring the horizontally transferred Copia sequence otherwise present in several other natural populations. Indeed, the sequenced strain of *D. willistoni*, Gd-H4-1, corresponds to a population from Guadeloupe Island (Caribbean) [7] that has not been tested in the work of [24]. A similar observation has been made in South American populations of *D. willistoni* in which no evidence of the HT of Copia was detected [27].

Globally, the pattern of species presence/absence in the phylogenetic tree displayed on Fig. 1 is compatible with a large majority of vertical transmissions for elements from the *Ty1/Copia* superfamily. Indeed, some TEs may have been lost in several of the analyzed species. For example, the lack of *Ty1/Copia* sequences in *D. erecta* was confirmed by the blastn searches using reference sequences from the other species from the *melanogaster* subgroup on its genome sequence (Additional file 4: Figure S2a). Some families are also absent from *D. yakuba* (1731, COPIA2bis, and Frogger, see Additional file 4: Figure S2a). The Copia-2_DYa from *D. yakuba* displayed hits of degraded fragments present in *D. melanogaster, D. simulans*, and *D. sechellia*, whereas the Copia-1_DY element is present in these species. Thus, it is likely that the first element was lost in the other species of the *melanogaster* subgroup, *D. yakuba* excepted.

#### The phylogenetic analysis of *BEL/Pao* reveals several cases of intra-species diversification and a majority of vertical transmissions among Drosophila families

This group has been shown to be reduced to metazoan species contrary to other LTR-retrotransposon groups, which suggests that it could have arisen early in the metazoan evolution [20, 28]. The families from this group are more numerous than those from the *Ty1/Copia* superfamily (Table 1). All species harbor several families from this superfamily, the species with the most numerous number of families being *D. ananassae*, *D. takahashii*, and *D. willistoni,* with respectively 22, 22 and 21 families*,* which is not particularly the case for the species from the *melanogaster* subgroup, which contain less than a dozen of families*.*

**Fig. 2:**
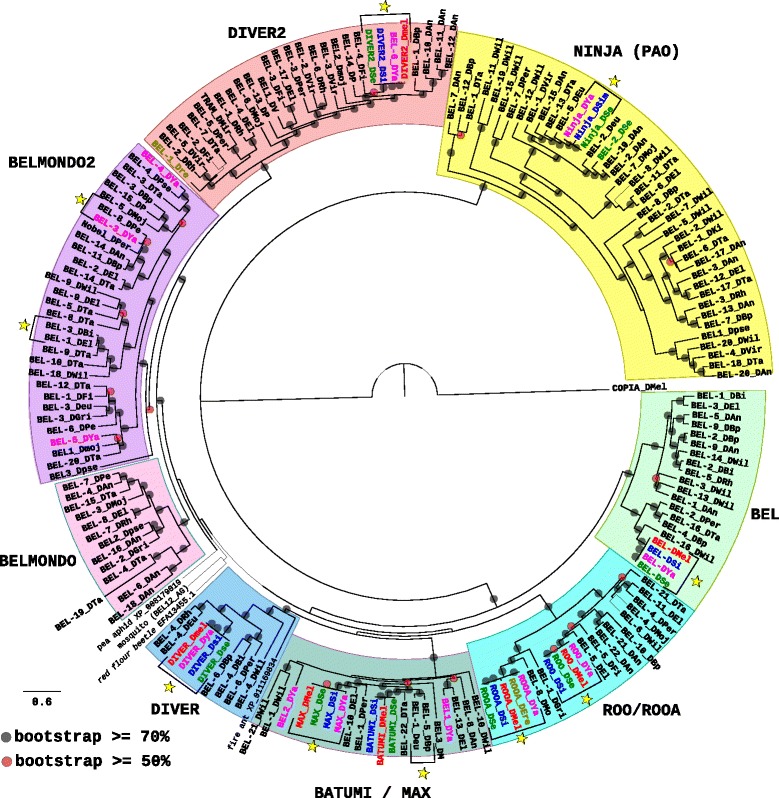
Maximum likelihood phylogenetic tree based on the polyprotein amino acid sequences of *BEL/Pao* elements. Only bootstrap values greater than 50% (*red dots*) and greater than 70% (*black dot*) are indicated. The tree has been rooted by the COPIA element from *D. melanogaster*. The names of the species are abbreviated as follows: DAn, *D. ananassae*; DBi, *D. biarmipes*; DBp, *D. bipectina*; DEl, *D. elegans;* DEre, *D. erecta* (*in yellow*); DEu/Deu*, D. eugracilis;* DFi, *D. ficusphila*; DGri, *D. grimshawi;* DKi, *D. kikkawai*; DMel, *D. melanogaster* (*in red*); DMir, *D. miranda*; DMoj/Dmoj/Dmo/DM, *D. mojavensis*; DPer/Dpe/DP, *D. persimilis*; DPse/Dpse, *D. pseudoobscura*; DRh, *D. rhopaloa*; DSe, *D. sechellia* (*in green*); DSi, *D. simulans* (*in blue*); DTa, *D. takahashi*; DVir/DV, *D. virilis*; DWil, *D. willistoni*; DYa, *D. yakuba* (*in pink*). Four sequences from other insects are included. *Yellow stars* represent cases of confirmed horizontal transfers (see details in Additional file 3: Figure S1b)

The phylogenetic tree based on the pol protein of these families (Fig. 2) allowed to distinguish the two main known clades BEL and PAO [20, 28] with high bootstrap value supports. The BEL clade can also be subdivided in several highly supported subclades among which DIVER2, DIVER, BATUMI/MAX, ROO/ROOA and BEL, which were already documented [28], and BELMONDO and BELMONDO2 representing two new subclades. Reference elements from these two new subclades are not present in the species from the *melanogaster* subgroup excepted three families present in *D. yakuba* that belong to the BELMONDO2 subclade. We checked by blastn searches whether the absence of homologous elements in the other species of the *melanogaster* subgroup was real or only the reflexion of unidentified complete reference sequences. We were able to detect traces of elements in the other species in the case of BEL-3_DYa and BEL-4_DYa (Additional file 4: Figure S2b) but for BEL-5_DYa, no homologous sequence is present in *D. melanogaster* and *D. sechellia*. Interestingly, *D. erecta* is often devoid of families present in the other species of the *melanogaster* subgroup (Batumi, BEL, DIVER, and DIVER2) or only remnants can be found in its genome (Max and Ninja). There are several cases of what could be considered as recent emergences of new families inside a species (Fig. 2). A recent emergence corresponds to a clade of several different families inside a given species. This indicates, like for paralogous genes inside host gene families, that diversification events appeared after speciation events, inside the considered species. All these events are restricted to three species corresponding to those with the highest number of families: *D. ananassae* (BEL-21_DAn and BEL-22_DAn; BEL-19_DAn and BEL-2_DAn; BEL-10_DAn, BEL-11_DAn and BEL-12_DAn; BEL-6_DAn and BEL-18_DAn), *D. willistoni* (BEL-11_DWil and BEL-19_DWil) and *D. takahashii* (BEL-4_DTa and BEL-19_DTa). These elements could correspond to sequence variants, as it was observed for several LTR-retrotransposons in *D. simulans* and *D. sechellia* [6].

Some cases of HTs have been verified and mainly concern species from the *melanogaster* subgroup. Indeed, we were able to validate recent HTs between *D. yakuba* and *D. simulans* (BEL, DIVER, DIVER2, and Max), between *D. melanogaster* and *D. sechellia* (BEL and DIVER), more ancient HTs between an ancestor of *D. yakuba* and an ancestor of *D. sechellia/D. simulans* (Ninja), and between ancestor of *D. yakuba* and an ancestor of *D. sechellia/D. simulans/D. melanogaster* (ROO and ROOA) (Additional file 3: Figure S1b). Some of these events have been previously documented concerning ROO, BEL, Max, DIVER, and DIVER2 [10, 14, 16, 17]. However, the HTs of Ninja between the ancestors of *D. yakuba* and *D. sechellia/D. simulans* and of ROOA between the ancestors of *D. yakuba* and the other four species of the *melanogaster* subgroup were not documented before. We also detected three new cases of HTs implicating *D. yakuba* and *D. persimilis* (BEL-8_DPer, and BEL-3_DYa elements), *D. biarmipes* and *D. elegans* (BEL-3_DBi and BEL-1_DEl elements), and *D. eugracilis* and *D. bipectinata* (BEL-1_DEu and BEL-5_DBp elements) (Additional file 3: Figure S1b; Fig. 2). Then, although we can confirm HT events for some of the elements from the *BEL/Pao* group, their number is not very important compared to the diversity of sequences present in these species.

All these results indicate that duplications and losses of elements but not HT may have been the main drivers of the evolution of this group of TEs to explain the observed phylogenetic patterns among the 20 Drosophila species.

#### The phylogenetic analyses of *Ty3/Gypsy* families underline a highly diversified group of families in which many HT are identified for elements the most closely related to retroviruses

Elements from the *Ty3/Gypsy* superfamily correspond to the most diversified and numerous TEs in the *Drosophila* genomes (Table 1). Their diversity is particularly striking in *D. willistoni*, *D. ananassae*, *D. elegans* and the species from the *melanogaster* subgroup (excepted *D. erecta*) where they correspond to between 41 and 61 different families. The *Ty3/Gypsy* elements, which are widely represented among the eukaryotes, are closely related to retroviruses, some of them being even considered as real retroviruses like the Gypsy element, or at least as endogenous retroviruses like the elements Tirant, ZAM and Idefix in *D. melanogaster* [29–32].

Several big groups have been identified among the *Ty3/Gypsy* superfamily of Drosophila [20]. They have in common to be different from the *Ty3/Gypsy* chromoviruses, which are present in plants, fungi and vertebrates. Since it is not currently possible to determine by the name of the families in the majority of the Drosophila species the group to which they belong, we first built a phylogenetic tree based on the pol proteins of all families to have an idea of the boundaries of these groups (Additional file 5: Figure S3). This allowed us to define three groups (Group 1 “OSVALDO/ULYSSES”, Group 2 “MICROPIA/SACCO”, and Group 3 “errantiviridae/412”) for which we built three separated phylogenetic trees in order to have more resolved nodes and high statistical bootstrap values.

**Fig. 3:**
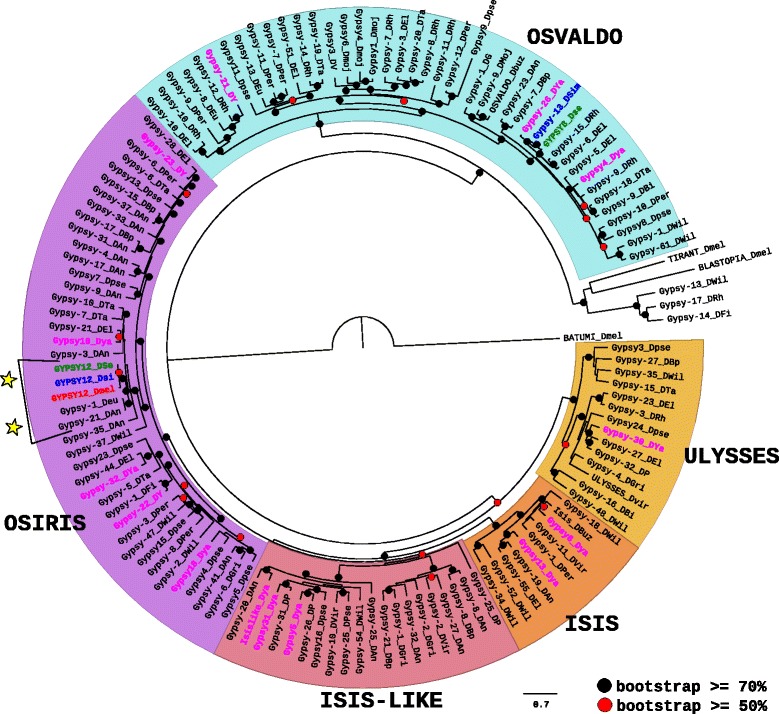
Maximum likelihood phylogenetic tree based on the polyprotein amino acid sequences of *Ty3/Gypsy* elements from the group “OSVALDO/ULYSSES”. Only bootstrap values greater than 50% (*red dots*) and greater than 70% (*black dot*) are indicated. The tree has been rooted by the Batumi element from *D. melanogaster* and we also added elements from the two other groups of *Ty3/Gypsy* (Tirant and BLASTOPIA from *D. melanogaster*). The names of the species are abbreviated as follows: DAn, *D. ananassae*; DBi, *D. biarmipes*; DBp, *D. bipectina*; Dbuz, *D. buzzatti*; DEl, *D. elegans;* DEu/Deu*, D. eugracilis;* DFi, *D. ficusphila*; DGri/DG, *D. grimshawi;* DKi, *D. kikkawai*; DMel, *D. melanogaster* (*in red*); DMoj/Dmoj, *D. mojavensis*; DPer/DP, *D. persimilis*; DPse/Dpse, *D. pseudoobscura*; DRh, *D. rhopaloa*; DSe, *D. sechellia* (*in green*); DSi, *D. simulans* (*in blue*); DTa, *D. takahashi*; DVir/DV, *D. virilis*; DWil, *D. willistoni*; DYa/DY, *D. yakuba* (*in pink*). *Yellow stars* represent cases of confirmed horizontal transfers (see details in Additional file 3: Figure S1c)

The phylogenetic tree based on the pol protein of families from the Group 1 “OSVALDO/ULYSSES” presents five main subgroups supported by strong bootstrap values (Fig. 3). Three of them correspond to already known clades (OSVALDO, ULYSSES, and ISIS), whereas the two last correspond to new clades (OSIRIS and ISIS-like). Originally, the elements Osvaldo [33] and Isis [34] were first described in *D. buzzati*, a species closely related to *D. mojavensis* from the repleta group, and Ulysses was first described in *D. virilis* from the same species complex [35]. This may explain why the species from the *melanogaster* subgroup are not well represented in Group 1 since only one family exists for *D. melanogaster* (GYPSY12), two for *D. sechellia* (GYPSY12_Dse and GYPSY6_Dse), two for *D. simulans* (GYPSY12_Dsi and Gypsy-13_Dsim), and none for *D. erecta*. However, *D. yakuba* possess 14 families, that are distributed among each of the clades, indicating that they do not originate from recent diversification inside this species. The majority of these elements are absent from the other species from the *melanogaster* subgroup, which could indicate that they have been lost in these last species (Additional file 4: Figure S2c). Indeed, the presence of these families in *D. yakuba* do not seem to be due to HT events, at least not with the species analyzed in this work. Then, these families could be present in *D. yakuba* since a long time. All other Drosophila species harbor more or less families from this group (from two in *D. biarmipes* and *D. fichusphila*, to 17 in *D. ananassae*) with the exception of *D. kikkawai* in which no family has been identified. The only two HT events that were confirmed in this analysis concern the GYPSY12 elements between *D. melanogaster* and *D. sechellia*, and the elements Gypsy-1_Deu and Gypsy-22_DAn between *D. eugracilis* and *D. ananassae* (Additional file 3: Figure S1c). Several cases of recent emergences of new families inside a species can be pointed out (Fig. 3). They concern *D. willistoni* (Gypsy-1_DWil and Gypsy-61_DWil in the OSALDO clade, Gypsy-52_DWil and Gypsy-34_DWil in the ISIS clade), *D. persimilis* (Gypsy-7_DPer and Gypsy-11_DPer in the OSIRIS clade), *D. ananassae* (Gypsy-4_DAn and Gypsy-17_DAn in the OSVALDO clade) and *D. mojavensis* (Gypsy1_Dmoj, Gypsy4_Dmoj and Gypsy6_Dmoj in the OSVALDO clade).

To summarize, the evolutionary history of the elements from the group “OSVALDO/ULYSSES” among the 20 Drosophila species seems to be mainly represented by vertical transmissions, with cases of intra-specific duplications and losses but almost no HT.

**Fig. 4:**
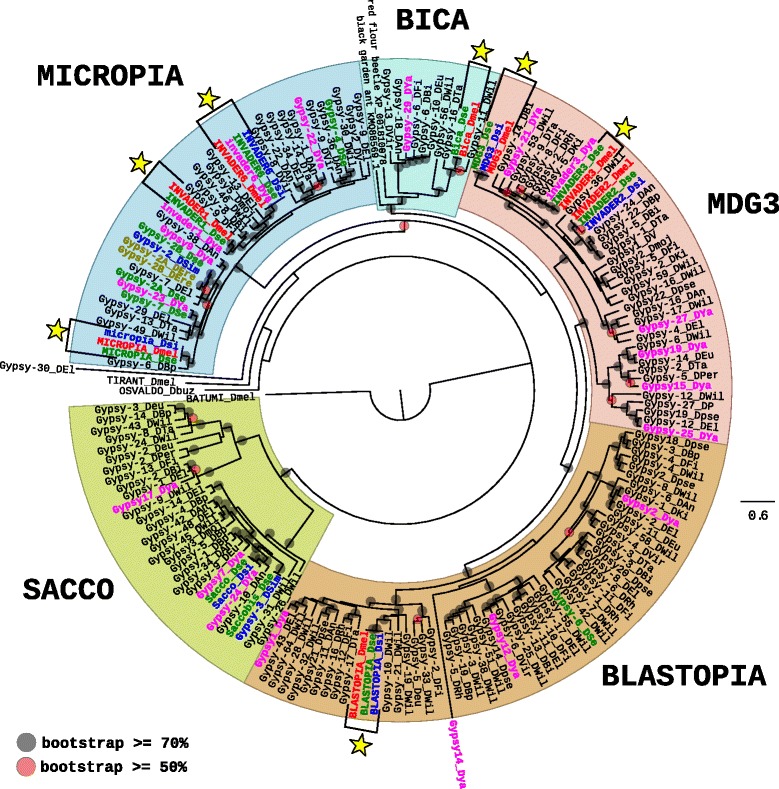
Maximum likelihood phylogenetic tree based on the polyprotein amino acid sequences of *Ty3/Gypsy* elements from the group “MICROPIA/SACCO”. Only bootstrap values greater than 50% (*red dots*) and greater than 70% (*black dot*) are indicated. The tree has been rooted by the Batumi element from *D. melanogaster* and we also added elements from the two other groups of *Ty3/Gypsy* (Tirant from *D. melanogaster* and Osvaldo from *D. buzzati*). The names of the species are abbreviated as follows: DAn, *D. ananassae*; DBi, *D. biarmipes*; DBp, *D. bipectina*; Dbuz, *D. buzzatti*; DEl, *D. elegans;* DEre, *D. erecta;* DEu/Deu*, D. eugracilis;* DFi, *D. ficusphila*; DMel, *D. melanogaster* (*in red*); Dmoj, *D. mojavensis*; DPer/DP, *D. persimilis*; Dpse, *D. pseudoobscura*; DRh, *D. rhopaloa*; DSe, *D. sechellia* (*in green*); DSi, *D. simulans* (*in blue*); DTa, *D. takahashi*; DVir/DV, *D. virilis*; DWil, *D. willistoni*; DYa/Dya, *D. yakuba* (*in pink*). Two sequences from other insects are included. *Yellow stars* represent cases of confirmed horizontal transfers (see details in Additional file 3: Figure S1d)

The Group 2, “MICROPIA/SACCO”, can be separated into two main clades in the phylogenetic tree based on the pol proteins (Fig. 4), one grouping elements of the MICROPIA/MDG3 type and the other corresponding to a new clade that we named SACCO. Two known subclades (BLASTOPIA and BICA), well supported by strong bootstrap values, are present near the two subclades MDG3 and MICROPIA. All Drosophila species harbor elements from this Group, *D. grimshawi* excepted. However, the number of families greatly varies from one species to another, going from two in *D. erecta*, to 35 in *D. willistoni*. In the species from the *melanogaster* subgroup, they are moderately numerous in *D. sechellia* and *D. yakuba* (with respectively 15 and 19 families) but less abundant in *D. melanogaster* and *D. simulans* (eight families in each). Interestingly, the families present in *D. yakuba* do not often have homologs in the other species from the *melanogaster* subgroup (Additional file 4: Figure S2c). Several families from *D. melanogaster* (seven families), *D. simulans* (four families), *D. sechellia* (seven families) and *D. yakuba* (two families) seem to be implicated in cases of HT events among the *melanogaster* subgroup (See yellow stars in the Fig. 4 and results from VHICA analyses displayed in Additional file 3: Figure S1c). Except the HT event concerning the blastopia element between *D. melanogaster* and *D. sechellia*, these HT events were already documented before [16, 17, 23, 36]. Among all the families, only one case of intra-specific diversification can be observed for the elements Gypsy-59_DWil and Gypsy-16_DWil.

Globally, elements from this group have mainly a history of vertical transmissions with few cases of HT identified that occurred between *D. melanogaster* and *D. sechellia*, and *D. yakuba* and *D. simulans.*

**Fig. 5:**
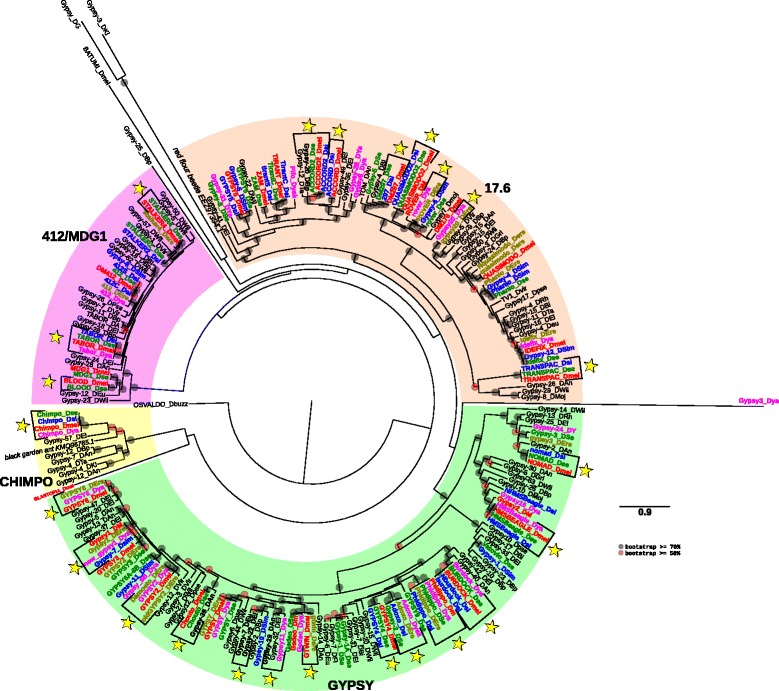
Maximum likelihood phylogenetic tree based on the polyprotein amino acid sequences of *Ty3/Gypsy* elements from the group “errantiviridae/412”. Only bootstrap values greater than 50% (*red dots*) and greater than 70% (*black dot*) are indicated. The tree has been rooted by the Batumi element from *D. melanogaster* and we also added elements from the two other groups of *Ty3/Gypsy* (Blastopia from *D. melanogaster* and Osvaldo from *D. buzzati*). The names of the species are abbreviated as follows: DAn, *D. ananassae*; DBi, *D. biarmipes*; DBp, *D. bipectina*; Dbuz, *D. buzzatti*; DEl, *D. elegans;* DEre, *D. erecta;* DEu/Deu*, D. eugracilis;* DFi, *D. ficusphila*; DGri/DG, *D. grimshawi*; DKi, *D. kikkawai*; Dmel/DM, *D. melanogaster* (*in red*); DMoj/Dmoj, *D. mojavensis*; Dpse, *D. pseudoobscura*; DRh, *D. rhopaloa*; Dse, *D. sechellia* (*in green*); Dsi, *D. simulans* (*in blue*); DTa, *D. takahashi*; DVir, *D. virilis*; DWil, *D. willistoni*; DY/Dya, *D. yakuba* (*in pink*). Two sequences from other insects are included. *Yellow stars* represent cases of confirmed horizontal transfers (see details in Additional file 3: Figure S1d and e)

The Group 3 “errantiviridae/412” is the largest of all three groups by the number of families present in the analyzed species. Only one species, *D. persimilis*, is devoid of elements from this type. The number of families is however quite variable, going from only one in *D. ficusphila* or two in *D. kikkawai* and *D. rhopoloa*, to 32 in *D. melanogaster* and *D. simulans* or 34 in *D. sechellia*. Some of the families present in the three last species are not always present in *D. yakuba* and *D. erecta* like for example ACCORD, Pifo, and QUASIMODO. The elements Gypsy-8_Dsim and Gypsy-5_DSe seem also to be usually absent from the other species of the *melanogaster* subgroup, excepted *D. simulans* (for Gypsy-5_DSe) and *D. sechellia* (for Gypsy-8_Dsim) (Additional file 4: Figure S2c). In these cases, it is likely that these elements have been lost in the species where they cannot be found. The phylogenetic tree based on the pol proteins displayed four known clades: CHIMPO, 412/MDG1, 17.6, and GYPSY (Fig. 5). Excepted in the CHIMPO clade, the species from the *melanogaster* subgroup possess several families inside each clade. A large number of families from these species are involved in HT events (see yellow stars in the Fig. 5 and Additional file 3: Figure S1d, e, and f). Several of them correspond to already described events in other works [6, 14, 16–18, 36, 37]. However, for some of the previously described elements involved in HTs among *D. melanogaster, D. simulans* and *D. yakuba* (Chimpo, Tabor and Chouto) we found that *D. sechellia* but also *D. ananassae* in the case of the Chouto element, may also be involved in HTs (Fig. 5, Additional file 3: Figure S1d, e, and f). We also detected new cases of HT events implicating species of the *melanogaster* subgroup like between *D. melanogaster* and *D. sechellia* (QUASIMODO2), *D. melanogaster* and *D. erecta* (gtwin), *D. melanogaster* and *D. yakuba* (Damoeto/GYPSY2), *D. yakuba* and *D. erecta* (gypsy20_Dya/gypsy20_DEre, rover and adoxo), *D. yakuba* and *D. ananassae* (Gypsy11_Dya/Gypsy-29_DAn). We also detected a case of HT between *D. elegans* and *D. eugracilis* (Gypsy-22_DEl and Gypsy-7_DEu), and between the ancestor of *D. bipectinata* and the ancestor of *D. melanogaster/D. simulans/D. sechellia* (ACCORD2/Gypsy-20_DBp). Based on the phylogenetic tree displayed in Fig. 5, we can observe four cases of intra-specific diversifications that happened in *D. willistoni* (Gypsy-5_DWil/Gypsy-50_DWil in the 412/MDG1 clade), and inside the GYPSY clade in *D. elegans* (Gypsy-47_DEl/Gypsy-20_DEl), in *D. ananassae* (Gypsy-5_DAn/Gypsy-13_DAn), and *D. bipectinata* (Gypsy-23_DBp/Gypsy-10_DBp).

In summary for the families of the group 3 “errantiviridae/412”, the evolutionary history of these elements seem to have implied a substantial amount of HTs at least among the species from the *melanogaster* subgroup but also a majority of vertical transmissions, losses and few intra-specific events of diversification in the other species. The elements from this group being the most similar to retroviruses compared to other LTR-retrotransposons, it is thus possible than they may be more prone for HT than other type of elements due to their capacity to form virus-like particles or by being in some cases infectious, as demonstrated in the *melanogaster* subgroup [38].

### Proportion of LTR retrotransposons is highly variable among the species but is not directly associated with genome size

**Fig. 6:**
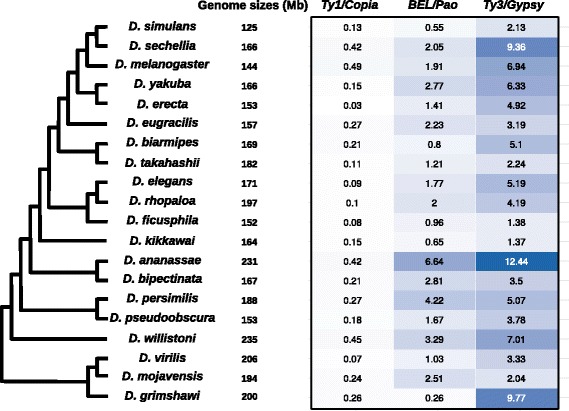
Proportion (in %) in the genomes of the 20 Drosophila species of each superfamily of LTR retrotransposons. The intensity of the *blue colors* is proportional to the TE proportion. The species are presented according to the phylogenetic tree topology as proposed by Seetharam & Stuart 2013, and we have indicated the genome sizes of each sequenced genome

We determined the proportion of each superfamily of LTR-retrotransposons in the assemblies of each species. The results are presented in the Fig. 6. We can see that for all species, elements from the *Ty1/Copia* superfamily are the less abundant, followed by the elements from the *BEL/Pao* superfamily, the elements from the *Ty3/Gypsy* superfamily being the most abundant. *D. ananassae* and *D. persimilis* are the species presenting the highest content of *BEL/Pao* elements with respectively 6.64 and 4.22%. Concerning the *Ty3/Gypsy* type elements, they are particularly abundant in *D. sechellia* (9.36%), *D. grimshawi* (9.77%) and *D. ananassae* (12.44%). In the case of the last species, the global abundance of LTR-retrotransposons (19.5% in total) is in agreement with the global estimate of repeats found in this assembly [7] or based on raw reads [39] although for this last estimate, the proportion of LTR-retrotransposons is lower that what we found. The values are more surprising in the case of *D. sechellia* and *D. grimshawi* for which the total estimate of repeat content originally described in the 12 genomes manuscript was quite low (respectively 3.67 and 2.84% [7]). Interestingly, in both cases, the proportion we observed is almost entirely attributable to only one family: Tabor in the case of *D. sechellia* (representing 3.15% of the genome) and Gypsy-5_DGri in the case of *D. grimshawi* (representing 8.62% of the genome). Since both families have been recently described (it is a new reference sequence described in this work for Tabor in *D. sechellia* and the one of *D. grimshawi* has been described in Repbase in 2011 after the publication of the genome sequence), it is possible that they were not detected by the initial TE annotation performed on the first assemblies in 2007.

For all the other species, we did not observe particular individual families with a high proportion. The global proportion of LTR-retrotransposons is rather the result of the cumulative sum of numerous different families. Indeed, we observed a significant positive correlation between the genome size (indicated in Fig. 6) and the number of references (i.e. the number of families as indicated in the last column of Table 1) present in a given species (Spearman correlation test *r* = 0.56, *p*-value = 0.01026). This is in contradiction with a previous work for which no correlation was observed between the genome size and the TE diversity among various eukaryotes [40]. However, in this last study, very distant organisms were considered going from fungi to animals, whereas in our case, all the considered species diverged at most 40 Myr ago. Interestingly, we did not observe a significant correlation between the genome size (Fig. 6) and the proportion of LTR-retrotransposons (Spearman correlation test *r* = 0.41, *p*-value = 0.07115) whereas it has been shown that the proportion of repeats is correlated with Drosophila genome size, in link with phylogenetic inertia [39]. It is possible that the lack of correlation comes from the fact that we are considering only LTR-retrotransposons. The non-LTR retrotransposons and DNA transposons can indeed represent significant proportions in some of the Drosophila species [7, 39].

## Conclusion

In this work, we have for the first time been able to replace all families of LTR-retrotransposons from 20 species of Drosophila in a phylogenetic framework, allowing to clearly determine to which group inside each superfamily they belong. This will allow more detailed analyses concerning the specific evolution of particular families in different species. Indeed, it will now be possible to look more closely at specific families displaying sequence variants in some species to try understand how they were formed. For that, further analyses need to be performed like phylogenetic analyses based on other parts of the elements than the pol protein. This should help us determine if recombination between TE families in some species, like *D. willistoni* or *D. ananassae*, may explain why the number of families is so high in their genomes for example.

We also confirmed that HT events may occur for LTR-retrotransposons, mainly among some species from the *melanogaster* subgroup, but that they do not represent the most usual way in the evolutionary dynamics of LTR-retrotransposons since vertical transmissions, but also losses and intra-specific diversification play a large role.

## Methods

### Genomic data

The fasta genome sequences from the 20 Drosophila species were retrieved from the flybase website (ftp://ftp.flybase.net/genomes/) for *D. ananassae* (v1.04), *D. erecta* (v1.04), *D. grimshawi* (v1.3), *D. melanogaster* (v6.05), *D. mojavensis* (v1.04), *D. persimilis* (v1.3), *D. pseudoobscura* (v3.2), *D. sechellia* (v1.3), *D. simulans* (v2.01), *D. virilis* (v1.2), *D. willistoni* (v1.3), and *D. yakuba* (v1.3), and from the NCBI website (http://www.ncbi.nlm.nih.gov) for *D. biarmipes* (v2.0), *D. bipectinata* (v2.0), *D. elegans* (v2.0), *D. eugracilis* (v2.0), *D. ficusphila* (v2.0), *D. kikkawai* (v2.0), *D. rhopoloa* (v2.0), and *D. takahashii* (v2.0). These genomes have been obtained using different sequencing technologies and have various levels of qualities concerning the sequencing coverage and the assembly effort [7].

### Identification of reference elements

Since *D. melanogaster* is a well annotated genome, we directly used the consensus sequences of LTR retrotransposons that are present in Repbase for this species [12]. To determine the reference elements of the other species from the *melanogaster* subgroup (*D. simulans, D. sechellia, D. yakuba*, and *D. erecta*), we first used the program LTRharvest [21] using the parameters settled for *D. melanogaster* since the program gave very good results for this species [22]. This program allows to identify potential complete LTR retrotransposons based on their structure. For each species, the candidates were then clustered using Uclust [41] with parameter -id 0.9. The sequences of each cluster were aligned using MUSCLE v3.8.31 [42] and the alignments were visualized with Seaview version 4.4.2 [43] to built a consensus for each cluster. Each consensus was manually corrected in regard to the other sequences to obtain a potentially “active” element with coding capacity. We also used each reference element to perform blastn [44] searches against the *D. simulans, D. sechellia, D. yakuba*, and *D. erecta* genomes to retrieve incomplete sequences of LTR retrotransposons not found by LTRharvest and to built consensus sequences using the alignments of the copies with low divergence compared to the reference sequence*.* We compared each reconstructed consensus with the sequences present in Repbase, Flybase and Genbank to identify already known elements and thus discriminating new characterized elements. We used the NCBI ORFfinder software (https://www.ncbi.nlm.nih.gov/orffinder/) to identify and retrieve the pol proteins. For the other 15 *Drosophila* species, we retrieved the consensus sequences corresponding to the internal part of the elements from Repbase and used ORFfinder to identify and retrieve the pol proteins. For 15 of them, either no coding capacity was detected or the corresponding gene was corresponding to gag or env, and they were thus not included in the phylogenetic reconstructions. In total for the 20 Drosophila species, we obtained 563 sequences from the *Ty3/Gypsy* superfamily, 195 from the *BEL/Pao* superfamily, and 67 from the *Ty1/Copia* superfamily.

We used the BLASTN facility of flybase (http://flybase.org/blast/) to determine the absence of elements in species for which no reference elements were identified but which were present in closely related species of the *melanogaster* subgroup. We considered an element as “present” if a reference was described in the species (either in this work or from previous works) or if we detected few very long hits with high sequence identity (>90%), as “absent” if the blast searches lead to either no match or not significant ones (small fragments of less than 100 bp), with “traces” if the blast searches lead to numerous significant fragmented matches with low sequence identity (<90%).

### Alignment and phylogenetic tree reconstruction

For each superfamily, *Ty3/Gypsy*, *BEL/Pao*, and *Ty1/Copia*, the protein sequences corresponding to pol of each reference element were aligned using MAFFT version 7 [45]. We added some sequences from a few other organisms available in Genbank (see figure legends). The non-informative sites in each alignment were removed using trimAL version 1.3 [46]. We determined the amino acid evolution model to be used in the phylogenetic reconstructions using ProtTest version 3 [47]. This analysis allowed us to reveal the same evolutionary model for protein evolution LG + I + G + F to best explain our data for each superfamily. Tree reconstructions were performed by maximum-likelihood method as implemented in PHYML 3.0 [48] with 100 bootstrap replicates using the LG + I + G + F evolutionary model. They were represented and edited using the FigTree software version 1.4.1 (Rambaut 2006–2013 http://tree.bio.ed.ac.uk/software/figtree/).

### Confirmation of LTR retrotransposon horizontal transfer (HT)

Phylogenetic incongruences of TEs clustered with homologs from distant drosophila species or very short branches grouping different species that could indicate HT events, were analyzed by using the VHICA method [23]. Briefly, this method is based on the differences between the evolution rate at synonymous positions between TEs and a set of vertically transferred reference genes but also taking into account the codon usage bias. For each compared pair of species, the correlation between the codon usage and the synonymous substitution rate is considered among reference genes assumed to be vertically transmitted. TEs with a significant deviation from host gene values are interpreted as potential horizontally transfered. To use VHICA, we performed the alignment of 30 orthologous genes among the 20 drosophila species using MACSE [49]. The list of the 30 genes correspond to a randomly selected subsample of the genes used by Wallau et al. [23] (Additional file 6: Table S2). The MACSE program was used to align the coding parts of the consensus TEs for which we had suspicion of HTs.

### Proportion of LTR retrotransposons in the 20 Drosophila genomes

The RepeatMasker program (Smit et al. 1996–2010 http://www.repeatmasker.org) was used on the complete genome sequences of the 20 drosophila using a custom library corresponding to all identified reference elements. The .out output file was then parsed using one_code_to_find_them_all [50] to determine the proportion of each superfamily.

## Additional files

Additional file 1: Fasta sequences of the newly described reference TEs. (FASTA 959 kb)

Additional file 2: Table S1. Repbase references (with internal part) for the all drosophila species with their associate superfamily and clade as found in the phylogenetic analyses (Figs. 1, 2, 3, 4 and 5). (PDF 311 kb)

Additional file 3: Figure S1. Graphical matrix view generated by the VHICA method for HT cases in the A) *Ty1/Copia* superfamily, B) BEL/Pao and C,D,E) *Ty3/Gypsy* superfamilies. (PDF 779 kb)

Additional file 4: Figure S2. Pattern of presence (black), absence (white) or traces (gray) of a given TE in the species of the *melanogaster* subgroup for A) Ty1/Copia, B) BEL/Pao, and C) Ty3/Gypsy superfamilies. (PDF 50 kb)

Additional file 5: Figure S3. Maximum likelihood treee based on the pot proteins of all Ty3/Gypsy elements. (PDF 35 kb)

Additional file 6: Table S2. Single copy orthologous genes from the Drosophila genomes used in the dS estimate in the VHICA method. (PDF 231 kb)

## Abbreviations

HT: Horizontal transfer
LTR: Long terminal repeat
TE: Transposable element
