# Supplementary material for: Evolutionary history of LTR-retrotransposons among 20 Drosophila species
Source: Mob DNA. 2017 Apr 27;8:7. doi: 10.1186/s13100-017-0090-3 (PMC5408442; doi:10.1186/s13100-017-0090-3)
Supplement: Supplementary file 2 — Repbase references (with internal part) for the all drosophila species with their associate superfamily and clade as found in the phylogenetic analyses (Figs. 1, 2, 3, 4 and 5). (PDF 311 kb) [file 13100_2017_90_MOESM2_ESM.pdf]

Supplementary Table S1: repbase references (with internal part) for the all drosophila species with their associate superfamily and clade as found in the phylogenetic analyses (figures 1 to 5)

**Reference name**	**Species**	**Superfamily**	**Clade**
BEL-1_DAn	*D. ananassae*	*BEL/Pao*	BEL
BEL-10_DAn	*D. ananassae*	*BEL/Pao*	DIVER2
BEL-11_DAn	*D. ananassae*	*BEL/Pao*	DIVER2
BEL-12_DAn	*D. ananassae*	*BEL/Pao*	DIVER2
BEL-13_DAn	*D. ananassae*	*BEL/Pao*	NINJA
BEL-14_DAn	*D. ananassae*	*BEL/Pao*	BELMONDO2
BEL-15_DAn	*D. ananassae*	*BEL/Pao*	NINJA
BEL-16_DAn	*D. ananassae*	*BEL/Pao*	BELMONDO
BEL-17_DAn	*D. ananassae*	*BEL/Pao*	NINJA
BEL-18_DAn	*D. ananassae*	*BEL/Pao*	BELMONDO
BEL-19_DAn	*D. ananassae*	*BEL/Pao*	NINJA
BEL-2_DAn	*D. ananassae*	*BEL/Pao*	NINJA
BEL-20_DAn	*D. ananassae*	*BEL/Pao*	NINJA
BEL-21_DAn	*D. ananassae*	*BEL/Pao*	ROO/ROOA
BEL-22_DAn	*D. ananassae*	*BEL/Pao*	ROO/ROOA
BEL-3_DAn	*D. ananassae*	*BEL/Pao*	NINJA
BEL-4_DAn	*D. ananassae*	*BEL/Pao*	BELMONDO
BEL-5_DAn	*D. ananassae*	*BEL/Pao*	BEL
BEL-6_DAn	*D. ananassae*	*BEL/Pao*	BELMONDO
BEL-7_DAn	*D. ananassae*	*BEL/Pao*	NINJA
BEL-8_DAn	*D. ananassae*	*BEL/Pao*	BATUMI / MAX
BEL-9_DAn	*D. ananassae*	*BEL/Pao*	BEL
Copia-1_DAn	*D. ananassae*	*Ty1/Copia*	COPIA
Copia-2_DAn	*D. ananassae*	*Ty1/Copia*	1731
Copia-3_DAn	*D. ananassae*	*Ty1/Copia*	COPIA
Copia-4_DAn	*D. ananassae*	*Ty1/Copia*	COPIA
Copia-5_DAn	*D. ananassae*	*Ty1/Copia*	COPIA
Copia-6_DAn	*D. ananassae*	*Ty1/Copia*	COPIA2_bis
Copia-7_DAn	*D. ananassae*	*Ty1/Copia*	Xanthias
Copia-8_DAn	*D. ananassae*	*Ty1/Copia*	COPIABIS
Gypsy-1_DAn	*D. ananassae*	*Ty3/Gypsy*	Group 2 – MICROPIA
Gypsy-10_DAn	*D. ananassae*	*Ty3/Gypsy*	Group 2 – SACCO
Gypsy-11_DAn	*D. ananassae*	*Ty3/Gypsy*	Group 2 – BLASTOPIA
Gypsy-12_DAn	*D. ananassae*	*Ty3/Gypsy*	Group 3 – CHIMPO
Gypsy-13_DAn	*D. ananassae*	*Ty3/Gypsy*	Group 3 – GYPSY
Gypsy-14_DAn	*D. ananassae*	*Ty3/Gypsy*	Group 3 – GYPSY
Gypsy-15_DAn	*D. ananassae*	*Ty3/Gypsy*	Group 3 – 17.6
Gypsy-16_DAn	*D. ananassae*	*Ty3/Gypsy*	Group 2 – MDG3

Gypsy-17_DAn	*D. ananassae*	*Ty3/Gypsy*	Group 1 – OSIRIS
Gypsy-18_DAn	*D. ananassae*	*Ty3/Gypsy*	Group 2 – BICA
Gypsy-19_DAn	*D. ananassae*	*Ty3/Gypsy*	Group 1 – ISIS
Gypsy-2_DAn	*D. ananassae*	*Ty3/Gypsy*	Group 3 – GYPSY
Gypsy-20_DAn	*D. ananassae*	*Ty3/Gypsy*	Group 1 – ISIS-like
Gypsy-21_DAn	*D. ananassae*	*Ty3/Gypsy*	Group 1 – OSIRIS
Gypsy-22_DAn	*D. ananassae*	*Ty3/Gypsy*	Group 2 – MICROPIA
Gypsy-23_DAn	*D. ananassae*	*Ty3/Gypsy*	Group 1 – OSVALDO
Gypsy-24_DAn	*D. ananassae*	*Ty3/Gypsy*	Group 2 – MDG3
Gypsy-25_DAn	*D. ananassae*	*Ty3/Gypsy*	Group 1 – ISIS
Gypsy-26_DAn	*D. ananassae*	*Ty3/Gypsy*	Group 3 – 17.6
Gypsy-27_DAn	*D. ananassae*	*Ty3/Gypsy*	Group 1 – ISIS-like
Gypsy-28_DAn	*D. ananassae*	*Ty3/Gypsy*	Group 3 – 412/MDG1
Gypsy-29_DAn	*D. ananassae*	*Ty3/Gypsy*	Group 3 – GYPSY
Gypsy-3_DAn	*D. ananassae*	*Ty3/Gypsy*	Group 1 – OSIRIS
Gypsy-30_DAn	*D. ananassae*	*Ty3/Gypsy*	Group 3 – GYPSY
Gypsy-31_DAn	*D. ananassae*	*Ty3/Gypsy*	Group 1 – OSIRIS
Gypsy-32_DAn	*D. ananassae*	*Ty3/Gypsy*	Group 1 – ISIS-like
Gypsy-33_DAn	*D. ananassae*	*Ty3/Gypsy*	Group 1 – OSIRIS
Gypsy-34_DAn	*D. ananassae*	*Ty3/Gypsy*	Group 2 – SACCO
Gypsy-35_DAn	*D. ananassae*	*Ty3/Gypsy*	Group 1 – OSIRIS
Gypsy-36_DAn	*D. ananassae*	*Ty3/Gypsy*	Group 2 – MICROPIA
Gypsy-37_DAn	*D. ananassae*	*Ty3/Gypsy*	Group 1 – OSIRIS
Gypsy-38_DAn	*D. ananassae*	*Ty3/Gypsy*	Group 2 – MICROPIA
Gypsy-39_DAn	*D. ananassae*	*Ty3/Gypsy*	Group 3 – GYPSY
Gypsy-4_DAn	*D. ananassae*	*Ty3/Gypsy*	Group 1 – OSIRIS
Gypsy-40_DAn	*D. ananassae*	*Ty3/Gypsy*	Group 3 – GYPSY
Gypsy-41_DAn	*D. ananassae*	*Ty3/Gypsy*	Group 1 – OSIRIS
Gypsy-42_DAn	*D. ananassae*	*Ty3/Gypsy*	Group 2 – SACCO
Gypsy-5_DAn	*D. ananassae*	*Ty3/Gypsy*	Group 3 – GYPSY
Gypsy-6_DAn	*D. ananassae*	*Ty3/Gypsy*	Group 2 – BLASTOPIA
Gypsy-7_DAn	*D. ananassae*	*Ty3/Gypsy*	Group 3 – CHIMPO
Gypsy-8_DAn	*D. ananassae*	*Ty3/Gypsy*	Group 1 – ISIS-like
Gypsy-9_DAn	*D. ananassae*	*Ty3/Gypsy*	Group 1 – OSIRIS
TABOR_DA	*D. ananassae*	*Ty3/Gypsy*	Group 3 – 412/MDG1
TOM_DAn	*D. ananassae*	*Ty3/Gypsy*	Group 3 – 17.6
BEL-1_DBi	*D. biarmipes*	*BEL/Pao*	BEL
BEL-2_DBi	*D. biarmipes*	*BEL/Pao*	BEL
BEL-3_DBi	*D. biarmipes*	*BEL/Pao*	BELMONDO2
BEL-4_DBi	*D. biarmipes*	*BEL/Pao*	DIVER
Copia-1_DBi	*D. biarmipes*	*Ty1/Copia*	COPIA
Gypsy-1_DBi	*D. biarmipes*	*Ty3/Gypsy*	Group 3 – 412/MDG1

Gypsy-11_DBi	*D. biarmipes*	*Ty3/Gypsy*	? (no pol)
Gypsy-13_DBi	*D. biarmipes*	*Ty3/Gypsy*	Group 2 – BLASTOPIA
Gypsy-14_DBi	*D. biarmipes*	*Ty3/Gypsy*	Group 2 – MDG3
Gypsy-15_DBi	*D. biarmipes*	*Ty3/Gypsy*	Group 3 – 17.6
Gypsy-16_DBi	*D. biarmipes*	*Ty3/Gypsy*	Group 1 – ULYSSES
Gypsy-17_DBi	*D. biarmipes*	*Ty3/Gypsy*	Group 3 – GYPSY
Gypsy-2_DBi	*D. biarmipes*	*Ty3/Gypsy*	Group 2 – SACCO
Gypsy-3_DBi	*D. biarmipes*	*Ty3/Gypsy*	Group 2 – BLASTOPIA
Gypsy-4_DBi	*D. biarmipes*	*Ty3/Gypsy*	Group 3 – 17.6
Gypsy-5_DBi	*D. biarmipes*	*Ty3/Gypsy*	Group 2 – MDG3
Gypsy-6_DBi	*D. biarmipes*	*Ty3/Gypsy*	Group 2 – BICA
Gypsy-7_DBi	*D. biarmipes*	*Ty3/Gypsy*	Group 3 – GYPSY
Gypsy-8_DBi	*D. biarmipes*	*Ty3/Gypsy*	Group 2 – MICROPIA
Gypsy-9_DBi	*D. biarmipes*	*Ty3/Gypsy*	Group 1 – OSVALDO
BEL-1_DBp	*D. bipectinata*	*BEL/Pao*	DIVER2
BEL-10_DBp	*D. bipectinata*	*BEL/Pao*	ROO/ROOA
BEL-11_DBp	*D. bipectinata*	*BEL/Pao*	BELMONDO2
BEL-12_DBp	*D. bipectinata*	*BEL/Pao*	NINJA
BEL-2_DBp	*D. bipectinata*	*BEL/Pao*	BEL
BEL-3_DBp	*D. bipectinata*	*BEL/Pao*	BELMONDO2
BEL-4_DBp	*D. bipectinata*	*BEL/Pao*	BEL
BEL-5_DBp	*D. bipectinata*	*BEL/Pao*	BATUMI / MAX
BEL-6_DBp	*D. bipectinata*	*BEL/Pao*	DIVER
BEL-7_DBp	*D. bipectinata*	*BEL/Pao*	NINJA
BEL-8_DBp	*D. bipectinata*	*BEL/Pao*	NINJA
BEL-9_DBp	*D. bipectinata*	*BEL/Pao*	BEL
Copia-1_DBp	*D. bipectinata*	*Ty1/Copia*	COPIA2/COPIA2_bis
Copia-2_DBp	*D. bipectinata*	*Ty1/Copia*	1731
Copia-3_DBp	*D. bipectinata*	*Ty1/Copia*	COPIABIS
Gypsy-1_DBp	*D. bipectinata*	*Ty3/Gypsy*	Group 1 – SACCO
Gypsy-10_DBp	*D. bipectinata*	*Ty3/Gypsy*	Group 3 – GYPSY
Gypsy-11_DBp	*D. bipectinata*	*Ty3/Gypsy*	Group 3 – 412/MDG1
Gypsy-12_DBp	*D. bipectinata*	*Ty3/Gypsy*	Group 3 – CHIMPO
Gypsy-13_DBp	*D. bipectinata*	*Ty3/Gypsy*	? (no pol)
Gypsy-14_DBp	*D. bipectinata*	*Ty3/Gypsy*	Group 1 – SACCO
Gypsy-15_DBp	*D. bipectinata*	*Ty3/Gypsy*	Group 2 – OSIRIS
Copia-1_DBp	*D. bipectinata*	*Ty3/Gypsy*	COPIA2/COPIA2_bis
Gypsy-16_DBp	*D. bipectinata*	*Ty3/Gypsy*	? (too short)
Gypsy-17_DBp	*D. bipectinata*	*Ty3/Gypsy*	Group 2 – OSIRIS
Gypsy-18_DBp	*D. bipectinata*	*Ty3/Gypsy*	Group 3 – 412/MDG1
Gypsy-19_DBp	*D. bipectinata*	*Ty3/Gypsy*	Group 1 – BLASTOPIA
Gypsy-2_DBp	*D. bipectinata*	*Ty3/Gypsy*	Group 3 – GYPSY

Gypsy-20_DBp	*D. bipectinata*	*Ty3/Gypsy*	Group 3 – 17.6
Gypsy-21_DBp	*D. bipectinata*	*Ty3/Gypsy*	Group 2 – ISIS-like
Gypsy-22_DBp	*D. bipectinata*	*Ty3/Gypsy*	Group 1 – MDG3
Gypsy-23_DBp	*D. bipectinata*	*Ty3/Gypsy*	Group 3 – GYPSY
Gypsy-24_DBp	*D. bipectinata*	*Ty3/Gypsy*	Group 3 – 17.6
Gypsy-25_DBp	*D. bipectinata*	*Ty3/Gypsy*	Group 3
Gypsy-26_DBp	*D. bipectinata*	*Ty3/Gypsy*	Group 3 – GYPSY
Gypsy-27_DBp	*D. bipectinata*	*Ty3/Gypsy*	Group 2 – ULYSSES
Gypsy-28_DBp	*D. bipectinata*	*Ty3/Gypsy*	Group 3 – 412/MDG1
Gypsy-3_DBp	*D. bipectinata*	*Ty3/Gypsy*	Group 1 – BLASTOPIA
Gypsy-4_DBp	*D. bipectinata*	*Ty3/Gypsy*	Group 1 – SACCO
Gypsy-5_DBp	*D. bipectinata*	*Ty3/Gypsy*	Group 1 – SACCO
Gypsy-6_DBp	*D. bipectinata*	*Ty3/Gypsy*	Group 1 – MICROPIA
Gypsy-7_DBp	*D. bipectinata*	*Ty3/Gypsy*	Group 2 – OSVALDO
Gypsy-8_DBp	*D. bipectinata*	*Ty3/Gypsy*	Group 2 – ISIS-like
Gypsy-9_DBp	*D. bipectinata*	*Ty3/Gypsy*	Group 3 – 17.6
BEL-1_DEl	*D. elegans*	*BEL/Pao*	BELMONDO2
BEL-10_DEl	*D. elegans*	*BEL/Pao*	BATUMI / MAX
BEL-11_DEl	*D. elegans*	*BEL/Pao*	ROO/ROOA
BEL-12_DEl	*D. elegans*	*BEL/Pao*	NINJA
BEL-13_DEl	*D. elegans*	*BEL/Pao*	BATUMI / MAX
BEL-14_DEl	*D. elegans*	*BEL/Pao*	BATUMI / MAX
BEL-15_DEl	*D. elegans*	*BEL/Pao*	DIVER2
BEL-16_DEl	*D. elegans*	*BEL/Pao*	BELMONDO2
BEL-17_DEl	*D. elegans*	*BEL/Pao*	DIVER2
BEL-18_DEl	*D. elegans*	*BEL/Pao*	BELMONDO2
BEL-2_DEl	*D. elegans*	*BEL/Pao*	BELMONDO2
BEL-3_DEl	*D. elegans*	*BEL/Pao*	BEL
BEL-4_DEl	*D. elegans*	*BEL/Pao*	DIVER2
BEL-5_DEl	*D. elegans*	*BEL/Pao*	ROO/ROOA
BEL-6_DEl	*D. elegans*	*BEL/Pao*	NINJA
BEL-8_DEl	*D. elegans*	*BEL/Pao*	BELMONDO
BEL-9_DEl	*D. elegans*	*BEL/Pao*	BELMONDO2
Copia-1_DEl	*D. elegans*	*Ty1/Copia*	COPIABIS
Copia-2_DEl	*D. elegans*	*Ty1/Copia*	COPIA
Gypsy-1_DEl	*D. elegans*	*Ty1/Copia*	Group 2 – SACCO
Gypsy-10_DEl	*D. elegans*	*Ty3/Gypsy*	Group 1 – OSVALDO
Gypsy-11_DEl	*D. elegans*	*Ty3/Gypsy*	Group 2 – BLASTOPIA
Gypsy-12_DEl	*D. elegans*	*Ty3/Gypsy*	Group 2 – MDG3
Gypsy-13_DEl	*D. elegans*	*Ty3/Gypsy*	Group 2 – MICROPIA
Gypsy-14_DEl	*D. elegans*	*Ty3/Gypsy*	Group 2 – SACCO
Gypsy-15_DEl	*D. elegans*	*Ty3/Gypsy*	Group 3 – 17.6

Gypsy-16_DEl	*D. elegans*	*Ty3/Gypsy*	Group 3 – 17.6
Gypsy-17_DEl	*D. elegans*	*Ty3/Gypsy*	Group 2 – BLASTOPIA
Gypsy-18_DEl	*D. elegans*	*Ty3/Gypsy*	Group 3 – 412/MDG1
Gypsy-19_DEl	*D. elegans*	*Ty3/Gypsy*	? (no pol)
Gypsy-2_DEl	*D. elegans*	*Ty3/Gypsy*	Group 2 – BLASTOPIA
Gypsy-20_DEl	*D. elegans*	*Ty3/Gypsy*	Group 3 – GYPSY
Gypsy-21_DEl	*D. elegans*	*Ty3/Gypsy*	Group 1 – OSIRIS
Gypsy-22_DEl	*D. elegans*	*Ty3/Gypsy*	Group 3 – GYPSY
Gypsy-23_DEl	*D. elegans*	*Ty3/Gypsy*	Group 1 – ULYSSES
Gypsy-24_DEl	*D. elegans*	*Ty3/Gypsy*	Group 3 – 412/MDG1
Gypsy-25_DEl	*D. elegans*	*Ty3/Gypsy*	Group 3 – GYPSY
Gypsy-26_DEl	*D. elegans*	*Ty3/Gypsy*	? (no pol)
Gypsy-27_DEl	*D. elegans*	*Ty3/Gypsy*	Group 1 – ULYSSES
Gypsy-28_DEl	*D. elegans*	*Ty3/Gypsy*	Group 1 – OSIRIS
Gypsy-29_DEl	*D. elegans*	*Ty3/Gypsy*	Group 2 – MICROPIA
Gypsy-3_DEl	*D. elegans*	*Ty3/Gypsy*	Group 1 – OSVALDO
Gypsy-30_DEl	*D. elegans*	*Ty3/Gypsy*	Group 2
Gypsy-31_DEl	*D. elegans*	*Ty3/Gypsy*	Group 3 – GYPSY
Gypsy-32_DEl	*D. elegans*	*Ty3/Gypsy*	Group 3 – GYPSY
Gypsy-33_DEl	*D. elegans*	*Ty3/Gypsy*	Group 3 – GYPSY
Gypsy-34_DEl	*D. elegans*	*Ty3/Gypsy*	Group 2 – MICROPIA
Gypsy-35_DEl	*D. elegans*	*Ty3/Gypsy*	Group 3 – 17.6
Gypsy-36_DEl	*D. elegans*	*Ty3/Gypsy*	? (too short)
Gypsy-37_DEl	*D. elegans*	*Ty3/Gypsy*	Group 3 – GYPSY
Gypsy-38_DEl	*D. elegans*	*Ty3/Gypsy*	? (too short)
Gypsy-4_DEl	*D. elegans*	*Ty3/Gypsy*	Group 2 – MDG3
Gypsy-40_DEl	*D. elegans*	*Ty3/Gypsy*	? (too short)
Gypsy-41_DEl	*D. elegans*	*Ty3/Gypsy*	Group 3 – 17.6
Gypsy-42_DEl	*D. elegans*	*Ty3/Gypsy*	Group 3 – GYPSY
Gypsy-43_DEl	*D. elegans*	*Ty3/Gypsy*	Group 2 – BLASTOPIA
Gypsy-44_DEl	*D. elegans*	*Ty3/Gypsy*	Group 1 – OSIRIS
Gypsy-45_DEl	*D. elegans*	*Ty3/Gypsy*	? (too short)
Gypsy-46_DEl	*D. elegans*	*Ty3/Gypsy*	? (too short)
Gypsy-47_DEl	*D. elegans*	*Ty3/Gypsy*	Group 3 – GYPSY
Gypsy-48_DEl	*D. elegans*	*Ty3/Gypsy*	Group 2 – SACCO
Gypsy-49_DEl	*D. elegans*	*Ty3/Gypsy*	Group 3 – 17.6
Gypsy-5_DEl	*D. elegans*	*Ty3/Gypsy*	Group 1 – OSVALDO
Gypsy-51_DEl	*D. elegans*	*Ty3/Gypsy*	Group 1 – OSVALDO
Gypsy-52_DEl	*D. elegans*	*Ty3/Gypsy*	? (to many N)
Gypsy-53_DEl	*D. elegans*	*Ty3/Gypsy*	? (no pol)
Gypsy-54_DEl	*D. elegans*	*Ty3/Gypsy*	? (too short)
Gypsy-55_DEl	*D. elegans*	*Ty3/Gypsy*	Group 1 – ISIS

Gypsy-57_DEl	*D. elegans*	*Ty3/Gypsy*	Group 3 – CHIMPO
Gypsy-6_DEl	*D. elegans*	*Ty3/Gypsy*	Group 1 – OSVALDO
Gypsy-7_DEl	*D. elegans*	*Ty3/Gypsy*	Group 2 – MICROPIA
Gypsy-8_DEl	*D. elegans*	*Ty3/Gypsy*	Group 2 – BLASTOPIA
Gypsy-9_DEl	*D. elegans*	*Ty3/Gypsy*	Group 2 – MICROPIA
BEL-1_DEre	*D. erecta*	*BEL/Pao*	DIVER2
BEL-1_Deu	*D. eugracilis*	*BEL/Pao*	BATUMI / MAX
BEL-2_Deu	*D. eugracilis*	*BEL/Pao*	NINJA
BEL-3_Deu	*D. eugracilis*	*BEL/Pao*	BELMONDO2
BEL-4_DEu	*D. eugracilis*	*BEL/Pao*	DIVER
BEL-5_DEu	*D. eugracilis*	*BEL/Pao*	NINJA
Copia-1_Deu	*D. eugracilis*	*Ty1/Copia*	new Xanthias
Copia-2_Deu	*D. eugracilis*	*Ty1/Copia*	1731
Copia-3_DEu	*D. eugracilis*	*Ty1/Copia*	1731
Gypsy-1_Deu	*D. eugracilis*	*Ty3/Gypsy*	Group 1 – OSIRIS
Gypsy-10_DEu	*D. eugracilis*	*Ty3/Gypsy*	Group 2 – BICA
Gypsy-11_DEu	*D. eugracilis*	*Ty3/Gypsy*	Group 2 – BLASTOPIA
Gypsy-12_DEu	*D. eugracilis*	*Ty3/Gypsy*	Group 3 – 412/MDG1
Gypsy-13_DEu	*D. eugracilis*	*Ty3/Gypsy*	Group 1 – OSVALDO
Gypsy-14_DEu	*D. eugracilis*	*Ty3/Gypsy*	Group 2 – MDG3
Gypsy-2_Deu	*D. eugracilis*	*Ty3/Gypsy*	Group 2 – SACCO
Gypsy-3_Deu	*D. eugracilis*	*Ty3/Gypsy*	Group 2 – SACCO
Gypsy-4_Deu	*D. eugracilis*	*Ty3/Gypsy*	Group 3 – 17.6
Gypsy-5_Deu	*D. eugracilis*	*Ty3/Gypsy*	Group 2 – BLASTOPIA
Gypsy-6_DEu	*D. eugracilis*	*Ty3/Gypsy*	Group 3 – GYPSY
Gypsy-7_DEu	*D. eugracilis*	*Ty3/Gypsy*	Group 3 – GYPSY
Gypsy-8_DEu	*D. eugracilis*	*Ty3/Gypsy*	Group 1 – OSVALDO
Gypsy-9_DEu	*D. eugracilis*	*Ty3/Gypsy*	Group 2 – SACCO
BEL-1_DFi	*D. ficusphila*	*BEL/Pao*	BELMONDO2
BEL-2_DFi	*D. ficusphila*	*BEL/Pao*	DIVER2
BEL-3_DFi	*D. ficusphila*	*BEL/Pao*	DIVER2
BEL-4_DFi	*D. ficusphila*	*BEL/Pao*	DIVER2
BEL-5_DFi	*D. ficusphila*	*BEL/Pao*	ROO/ROOA
Copia-1_DFi	*D. ficusphila*	*Ty1/Copia*	1731
Gypsy-1_DFi	*D. ficusphila*	*Ty3/Gypsy*	Group 1 – OSIRIS
Gypsy-10_DFi	*D. ficusphila*	*Ty3/Gypsy*	Group 2 – BLASTOPIA
Gypsy-11_DFi	*D. ficusphila*	*Ty3/Gypsy*	Group 2 – BLASTOPIA
Gypsy-12_DFi	*D. ficusphila*	*Ty3/Gypsy*	Group 2 – BLASTOPIA
Gypsy-13_DFi	*D. ficusphila*	*Ty3/Gypsy*	Group 2 – SACCO
Gypsy-14_DFi	*D. ficusphila*	*Ty3/Gypsy*	Group 1
Gypsy-2_DFi	*D. ficusphila*	*Ty3/Gypsy*	Group 2 – BLASTOPIA
Gypsy-3_DFi	*D. ficusphila*	*Ty3/Gypsy*	? (too short)

Gypsy-4_DFi	*D. ficusphila*	*Ty3/Gypsy*	Group 2 – BLASTOPIA
Gypsy-5_DFi	*D. ficusphila*	*Ty3/Gypsy*	Group 2 – MDG3
Gypsy-6_DFi	*D. ficusphila*	*Ty3/Gypsy*	Group 2 – BICA
Gypsy-7_DFi	*D. ficusphila*	*Ty3/Gypsy*	Group 3 – GYPSY
Gypsy-8_DFi	*D. ficusphila*	*Ty3/Gypsy*	Group 2 – MDG3
Gypsy-9_DFi	*D. ficusphila*	*Ty3/Gypsy*	Group 2 – MICROPIA
BEL-1_DGri	*D. grimshawi*	*BEL/Pao*	ROO/ROOA
BEL-2_DGri	*D. grimshawi*	*BEL/Pao*	BELMONDO
BEL-3_DGri	*D. grimshawi*	*BEL/Pao*	BELMONDO2
Copia-1_DGri	*D. grimshawi*	*Ty1/Copia*	COPIABIS
Copia-2_DGri	*D. grimshawi*	*Ty1/Copia*	COPIABIS
Copia-3_DGri	*D. grimshawi*	*Ty1/Copia*	COPIA2/COPIA2_bis
Copia-4_DGri	*D. grimshawi*	*Ty1/Copia*	Outgroup
Gypsy_DG	*D. grimshawi*	*Ty3/Gypsy*	Group 3
Gypsy-1_DG	*D. grimshawi*	*Ty3/Gypsy*	Group 1 – OSVALDO
Gypsy-1_DGri	*D. grimshawi*	*Ty3/Gypsy*	Group 1 – ISIS-like
Gypsy-2_DGri	*D. grimshawi*	*Ty3/Gypsy*	Group 1 – ISIS
Gypsy-3_DGri	*D. grimshawi*	*Ty3/Gypsy*	Group 3 – 17.6
Gypsy-4_DGri	*D. grimshawi*	*Ty3/Gypsy*	Group 1 – ULYSSES
Gypsy-5_DGri	*D. grimshawi*	*Ty3/Gypsy*	Group 3 – GYPSY
Gypsy-6_DGri	*D. grimshawi*	*Ty3/Gypsy*	Group 1 – OSIRIS
BEL-1_DKi	*D. kikkawai*	*BEL/Pao*	NINJA
Copia-2_DKi	*D. kikkawai*	*Ty1/Copia*	COPIA/COPIABIS
Gypsy-1_DKi	*D. kikkawai*	*Ty3/Gypsy*	Group 2 – BLASTOPIA
Gypsy-2_DKi	*D. kikkawai*	*Ty3/Gypsy*	Group 2 – MDG3
Gypsy-3_DKi	*D. kikkawai*	*Ty3/Gypsy*	Group 3
Gypsy-4_DKi	*D. kikkawai*	*Ty3/Gypsy*	Group 3 – CHIMPO
Gypsy-5_DKi	*D. kikkawai*	*Ty3/Gypsy*	Group 2 – MDG3
ACCORD_Dmel	*D. melanogaster*	*Ty3/Gypsy*	Group 3 – 17.6
ACCORD2_Dmel	*D. melanogaster*	*Ty3/Gypsy*	Group 3 – 17.6
BATUMI_Dmel	*D. melanogaster*	*BEL/Pao*	BATUMI / MAX
BEL_DMel	*D. melanogaster*	*BEL/Pao*	BEL
Bica_Dmel	*D. melanogaster*	*Ty3/Gypsy*	Group 2 – BICA
BLASTOPIA_Dmel	*D. melanogaster*	*Ty3/Gypsy*	Group 2 – BLASTOPIA
BLOOD_Dmel	*D. melanogaster*	*Ty3/Gypsy*	Group 3 – 412/MDG1
BURDOCK_Dmel	*D. melanogaster*	*Ty3/Gypsy*	Group 3 – GYPSY
Chimpo_Dmel	*D. melanogaster*	*Ty3/Gypsy*	Group 3 – CHIMPO
Chouto_Dmel	*D. melanogaster*	*Ty3/Gypsy*	Group 3 – GYPSY
COPIA_Dmel	*D. melanogaster*	*Ty1/Copia*	COPIA
Copia-1_DM = Xanthias_Dmel	*D. melanogaster*	*Ty1/Copia*	Xanthias
COPIA2_Dmel	*D. melanogaster*	*Ty1/Copia*	COPIA2
DIVER_DMel	*D. melanogaster*	*BEL/Pao*	DIVER

DIVER2_DMel	*D. melanogaster*	*BEL/Pao*	DIVER2
DM1731_DMel	*D. melanogaster*	*Ty1/Copia*	1731
DM176_Dmel	*D. melanogaster*	*Ty3/Gypsy*	Group 3 – 17.6
DM297_Dmel	*D. melanogaster*	*Ty3/Gypsy*	Group 3 – 17.6
DM412_Dmel	*D. melanogaster*	*Ty3/Gypsy*	Group 3 – 412/MDG1
FROGGER_Dmel	*D. melanogaster*	*Ty3/Gypsy*	Xanthias
GTWIN_Dmel	*D. melanogaster*	*Ty3/Gypsy*	Group 3 – GYPSY
GYPSY_Dmel	*D. melanogaster*	*Ty3/Gypsy*	Group 3 – GYPSY
Gypsy1_DM	*D. melanogaster*	*Ty3/Gypsy*	Group 3 – GYPSY
GYPSY10	*D. melanogaster*	*Ty3/Gypsy*	? (no pol)
GYPSY11	*D. melanogaster*	*Ty3/Gypsy*	? (no pol)
GYPSY12_Dmel	*D. melanogaster*	*Ty3/Gypsy*	Group 1 – OSIRIS
GYPSY2_Dmel	*D. melanogaster*	*Ty3/Gypsy*	Group 3 – GYPSY
Gypsy2_DM	*D. melanogaster*	*Ty3/Gypsy*	Group 3 – GYPSY
GYPSY3_Dmel	*D. melanogaster*	*Ty3/Gypsy*	Group 3 – GYPSY
GYPSY4_Dmel	*D. melanogaster*	*Ty3/Gypsy*	Group 3 – GYPSY
GYPSY5_Dmel	*D. melanogaster*	*Ty3/Gypsy*	Group 3 – 17.6
GYPSY6_Dmel	*D. melanogaster*	*Ty3/Gypsy*	Group 3 – GYPSY
GYPSY7	*D. melanogaster*	*Ty3/Gypsy*	? (no pol)
GYPSY8	*D. melanogaster*	*Ty3/Gypsy*	? (no pol)
GYPSY9	*D. melanogaster*	*Ty3/Gypsy*	? (no pol)
HMSBEAGLE_Dmel	*D. melanogaster*	*Ty3/Gypsy*	Group 3 – GYPSY
IDEFIX_Dmel	*D. melanogaster*	*Ty3/Gypsy*	Group 3 – 17.6
INVADER1_Dmel	*D. melanogaster*	*Ty3/Gypsy*	Group 2 – MICROPIA
INVADER2_Dmel	*D. melanogaster*	*Ty3/Gypsy*	Group 2 – MDG3
INVADER3_Dmel	*D. melanogaster*	*Ty3/Gypsy*	Group 2 – MDG3
INVADER4_Dmel	*D. melanogaster*	*Ty3/Gypsy*	? (no pol)
INVADER5_Dmel	*D. melanogaster*	*Ty3/Gypsy*	? (no pol)
INVADER6_Dmel	*D. melanogaster*	*Ty3/Gypsy*	Group 1 – MICROPIA
MAX_DMel	*D. melanogaster*	*BEL/Pao*	BATUMI / MAX
MDG1_Dmel	*D. melanogaster*	*Ty3/Gypsy*	Group 3 – 412/MDG1
MDG3_Dmel	*D. melanogaster*	*Ty3/Gypsy*	Group 2 – MDG3
MICROPIA_Dmel	*D. melanogaster*	*Ty3/Gypsy*	Group 2 – MICROPIA
NOMAD_Dmel	*D. melanogaster*	*Ty3/Gypsy*	Group 3 – GYPSY
QUASIMODO_Dmel	*D. melanogaster*	*Ty3/Gypsy*	Group 3 – 17.6
QUASIMODO2_Dmel	*D. melanogaster*	*Ty3/Gypsy*	Group 3 – 17.6
ROO_DMel	*D. melanogaster*	*BEL/Pao*	ROO/ROOA
ROOA_DMel	*D. melanogaster*	*BEL/Pao*	ROO/ROOA
ROVER_DM	*D. melanogaster*	*Ty3/Gypsy*	Group 3 – 17.6
STALKER2_Dmel	*D. melanogaster*	*Ty3/Gypsy*	Group 3 – 412/MDG1
STALKER4_Dmel	*D. melanogaster*	*Ty3/Gypsy*	Group 3 – 412/MDG1
TABOR_Dmel	*D. melanogaster*	*Ty3/Gypsy*	Group 3 – 412/MDG1

TIRANT_Dmel	*D. melanogaster*	*Ty3/Gypsy*	Group 3 – 17.6
TRANSPAC_Dmel	*D. melanogaster*	*Ty3/Gypsy*	Group 3 – 17.6
ZAM_Dmel	*D. melanogaster*	*Ty3/Gypsy*	Group 3 – 17.6
BEL-3_DMoj	*D. mojavensis*	*BEL/Pao*	BELMONDO
BEL-4_DMoj	*D. mojavensis*	*BEL/Pao*	ROO/ROOA
BEL-5_DMoj	*D. mojavensis*	*BEL/Pao*	BELMONDO2
BEL-6_DMoj	*D. mojavensis*	*BEL/Pao*	DIVER2
BEL-7_DMoj	*D. mojavensis*	*BEL/Pao*	NINJA
BEL-8_DMoj	*D. mojavensis*	*BEL/Pao*	ROO/ROOA
BEL1_DMoj	*D. mojavensis*	*BEL/Pao*	BELMONDO2
BEL2_DMoj	*D. mojavensis*	*BEL/Pao*	DIVER2
BEL3_DMoj	*D. mojavensis*	*BEL/Pao*	BATUMI / MAX
Copia-3_DMoj	*D. mojavensis*	*Ty1/Copia*	1731
Copia-4_DMoj	*D. mojavensis*	*Ty1/Copia*	COPIA
Copia1_Dmoj	*D. mojavensis*	*Ty1/Copia*	outsider
Copia2_Dmoj	*D. mojavensis*	*Ty1/Copia*	COPIA
Gypsy-10_DMoj	*D. mojavensis*	*Ty3/Gypsy*	Group 3 – GYPSY
Gypsy-8_DMoj	*D. mojavensis*	*Ty3/Gypsy*	Group 3 – 17.6
Gypsy-9_DMoj	*D. mojavensis*	*Ty3/Gypsy*	Group 1 – OSVALDO
Gypsy1_Dmoj	*D. mojavensis*	*Ty3/Gypsy*	Group 1 – OSVALDO
Gypsy2_Dmoj	*D. mojavensis*	*Ty3/Gypsy*	Group 2 – MDG3
Gypsy3_Dmoj	*D. mojavensis*	*Ty3/Gypsy*	Group 2 – SACCO
Gypsy4_Dmoj	*D. mojavensis*	*Ty3/Gypsy*	Group 1 – OSVALDO
Gypsy5_Dmoj	*D. mojavensis*	*Ty3/Gypsy*	Group 2 – MICROPIA
Gypsy6_Dmoj	*D. mojavensis*	*Ty3/Gypsy*	Group 1 – OSVALDO
Gypsy7_Dmoj	*D. mojavensis*	*Ty3/Gypsy*	Group 3 – 17.6
BEL-1_DPer	*D. persimilis*	*BEL/Pao*	BATUMI / MAX
BEL-13_DP	*D. persimilis*	*BEL/Pao*	DIVER2
BEL-14_DP	*D. persimilis*	*BEL/Pao*	DIVER2
BEL-15_DP	*D. persimilis*	*BEL/Pao*	BELMONDO2
BEL-2_DPer	*D. persimilis*	*BEL/Pao*	BEL
BEL-3_DPer	*D. persimilis*	*BEL/Pao*	DIVER2
BEL-4_DPer	*D. persimilis*	*BEL/Pao*	ROO/ROOA
BEL-5_DPer	*D. persimilis*	*BEL/Pao*	DIVER2
BEL-6_DPe	*D. persimilis*	*BEL/Pao*	BELMONDO2
BEL-6_DPer	*D. persimilis*	*BEL/Pao*	DIVER
BEL-7_DPe	*D. persimilis*	*BEL/Pao*	BELMONDO
BEL-7_DPer	*D. persimilis*	*BEL/Pao*	NINJA
BEL-8_DPe	*D. persimilis*	*BEL/Pao*	BELMONDO2
Nobel_DPer	*D. persimilis*	*BEL/Pao*	BELMONDO2
Copia-1_DPer	*D. persimilis*	*Ty1/Copia*	COPIA
Copia-2_DPer	*D. persimilis*	*Ty1/Copia*	1731

Copia-29_DP	*D. persimilis*	*Ty1/Copia*	Xanthias
Gypsy-1_DPer	*D. persimilis*	*Ty3/Gypsy*	Group 1 – ISIS
Gypsy-10_DPer	*D. persimilis*	*Ty3/Gypsy*	Group 1 – OSVALDO
Gypsy-11_DPer	*D. persimilis*	*Ty3/Gypsy*	Group 1 – OSVALDO
Gypsy-12_DPer	*D. persimilis*	*Ty3/Gypsy*	Group 1 – OSVALDO
Gypsy-2_DPer	*D. persimilis*	*Ty3/Gypsy*	Group 2 – SACCO
Gypsy-25_DP	*D. persimilis*	*Ty3/Gypsy*	Group 1 – ISIS-like
Gypsy-26_DP	*D. persimilis*	*Ty3/Gypsy*	Group 1 – ISIS-like
Gypsy-27_DP	*D. persimilis*	*Ty3/Gypsy*	Group 2 – MDG3
Gypsy-3_DPer	*D. persimilis*	*Ty3/Gypsy*	Group 1 – OSIRIS
Gypsy-31_DP	*D. persimilis*	*Ty3/Gypsy*	Group 1 – ISIS-like
Gypsy-32_DP	*D. persimilis*	*Ty3/Gypsy*	Group 1 – ULYSSE
Gypsy-4_Dper	*D. persimilis*	*Ty3/Gypsy*	? (no pol)
Gypsy-5_DPer	*D. persimilis*	*Ty3/Gypsy*	Group 2 – MDG3
Gypsy-6_DPer	*D. persimilis*	*Ty3/Gypsy*	Group 1 – OSIRIS
Gypsy-7_DPer	*D. persimilis*	*Ty3/Gypsy*	Group 1 – OSVALDO
Gypsy-8_DPer	*D. persimilis*	*Ty3/Gypsy*	Group 1 – OSIRIS
Gypsy-9_DPer	*D. persimilis*	*Ty3/Gypsy*	Group 1 – OSVALDO
BEL-4_DPse	*D. pseudoobscura*	*BEL/Pao*	BELMONDO2
BEL1_Dpse	*D. pseudoobscura*	*BEL/Pao*	NINJA
BEL2_Dpse	*D. pseudoobscura*	*BEL/Pao*	BELMONDO
BEL3_DPse	*D. pseudoobscura*	*BEL/Pao*	BELMONDO2
Copia1_Dpse	*D. pseudoobscura*	*Ty1/Copia*	Xanthias
Copia2_Dpse	*D. pseudoobscura*	*Ty1/Copia*	COPIA2/COPIA2_bis/New Xanthias
Gypsy-25_DPse	*D. pseudoobscura*	*Ty3/Gypsy*	Group 1 – ISIS-like
Gypsy-26_DPse	*D. pseudoobscura*	*Ty3/Gypsy*	Group 3 – 412/MDG1
Gypsy1_Dpse	*D. pseudoobscura*	*Ty3/Gypsy*	? (no pol)
Gypsy10_Dpse	*D. pseudoobscura*	*Ty3/Gypsy*	Group 3 – GYPSY
Gypsy11_Dpse	*D. pseudoobscura*	*Ty3/Gypsy*	Group 1 – OSVALDO
Gypsy12_Dpse	*D. pseudoobscura*	*Ty3/Gypsy*	Group 3 – GYPSY
Gypsy13_Dpse	*D. pseudoobscura*	*Ty3/Gypsy*	Group 1 – OSIRIS
Gypsy14_Dpse	*D. pseudoobscura*	*Ty3/Gypsy*	Group 2 – BLASTOPIA
Gypsy15_Dpse	*D. pseudoobscura*	*Ty3/Gypsy*	Group 1 – OSIRIS
Gypsy16_Dpse	*D. pseudoobscura*	*Ty3/Gypsy*	Group 1 – ISIS-like
Gypsy17_Dpse	*D. pseudoobscura*	*Ty3/Gypsy*	Group 3 – 17.6
Gypsy18_Dpse	*D. pseudoobscura*	*Ty3/Gypsy*	Group 2 – BLASTOPIA
Gypsy19_Dpse	*D. pseudoobscura*	*Ty3/Gypsy*	Group 2 – MDG3
Gypsy2_Dpse	*D. pseudoobscura*	*Ty3/Gypsy*	Group 2 – BLASTOPIA
Gypsy20_Dpse	*D. pseudoobscura*	*Ty3/Gypsy*	Group 2 – BLASTOPIA
Gypsy21_Dpse	*D. pseudoobscura*	*Ty3/Gypsy*	Group 2 – BLASTOPIA
Gypsy22_Dpse	*D. pseudoobscura*	*Ty3/Gypsy*	Group 2 – MDG3

Gypsy23_Dpse	*D. pseudoobscura*	*Ty3/Gypsy*	Group 1 – OSIRIS
Gypsy24_Dpse	*D. pseudoobscura*	*Ty3/Gypsy*	Group 1 – ULYSSES
Gypsy3_Dpse	*D. pseudoobscura*	*Ty3/Gypsy*	Group 1 – ULYSSES
Gypsy4_Dpse	*D. pseudoobscura*	*Ty3/Gypsy*	Group 1 – OSIRIS
Gypsy5_Dpse	*D. pseudoobscura*	*Ty3/Gypsy*	Group 1 – OSIRIS
Gypsy6_Dpse	*D. pseudoobscura*	*Ty3/Gypsy*	Group 3 – GYPSY
Gypsy7_Dpse	*D. pseudoobscura*	*Ty3/Gypsy*	Group 1 – OSIRIS
Gypsy8_Dpse	*D. pseudoobscura*	*Ty3/Gypsy*	Group 1 – OSVALDO
Gypsy9_Dpse	*D. pseudoobscura*	*Ty3/Gypsy*	Group 1 – OSVALDO
BEL-1_DRh	*D. rhopaloa*	*BEL/Pao*	DIVER2
BEL-2_DRh	*D. rhopaloa*	*BEL/Pao*	DIVER2
BEL-3_DRh	*D. rhopaloa*	*BEL/Pao*	NINJA
BEL-4_DRh	*D. rhopaloa*	*BEL/Pao*	DIVER
BEL-5_DRh	*D. rhopaloa*	*BEL/Pao*	BEL
BEL-6_DRh	*D. rhopaloa*	*BEL/Pao*	DIVER2
BEL-7_DRh	*D. rhopaloa*	*BEL/Pao*	BELMONDO
Gypsy-1_DRh	*D. rhopaloa*	*Ty3/Gypsy*	Group 2 – BLASTOPIA
Gypsy-10_DRh	*D. rhopaloa*	*Ty3/Gypsy*	Group 1 – OSVALDO
Gypsy-11_DRh	*D. rhopaloa*	*Ty3/Gypsy*	Group 1 – OSVALDO
Gypsy-12_DRh	*D. rhopaloa*	*Ty3/Gypsy*	Group 1 – OSVALDO
Gypsy-13_DRh	*D. rhopaloa*	*Ty3/Gypsy*	Group 3 – GYPSY
Gypsy-14_DRh	*D. rhopaloa*	*Ty3/Gypsy*	Group 1 – OSVALDO
Gypsy-15_DRh	*D. rhopaloa*	*Ty3/Gypsy*	Group 1 – OSVALDO
Gypsy-16_DRh	*D. rhopaloa*	*Ty3/Gypsy*	Group 2 – BLASTOPIA
Gypsy-17_DRh	*D. rhopaloa*	*Ty3/Gypsy*	Group 1
Gypsy-2_DRh	*D. rhopaloa*	*Ty3/Gypsy*	Group 2 – MDG3
Gypsy-3_DRh	*D. rhopaloa*	*Ty3/Gypsy*	Group 1 – ULYSSES
Gypsy-4_DRh	*D. rhopaloa*	*Ty3/Gypsy*	Group 3 – 17.6
Gypsy-5_DRh	*D. rhopaloa*	*Ty3/Gypsy*	Group 2 – BLASTOPIA
Gypsy-6_DRh	*D. rhopaloa*	*Ty3/Gypsy*	Group 2 – BLASTOPIA
Gypsy-7_DRh	*D. rhopaloa*	*Ty3/Gypsy*	Group 1 – OSVALDO
Gypsy-8_DRh	*D. rhopaloa*	*Ty3/Gypsy*	Group 1 – OSVALDO
Gypsy-9_DRh	*D. rhopaloa*	*Ty3/Gypsy*	Group 1 – OSVALDO
BEL-2_DSe	*D. sechellia*	*BEL/Pao*	NINJA
Copia-1_DSe	*D. sechellia*	*Ty1/Copia*	COPIA2_bis
Gypsy-1_DSe	*D. sechellia*	*Ty3/Gypsy*	Group 3 – GYPSY
Gypsy-2_DSe	*D. sechellia*	*Ty3/Gypsy*	Group 3 – GYPSY
Gypsy-3_DSe	*D. sechellia*	*Ty3/Gypsy*	Group 3 – GYPSY
Gypsy-4_DSe	*D. sechellia*	*Ty3/Gypsy*	Group 2 – MICROPIA
Gypsy-5_DSe	*D. sechellia*	*Ty3/Gypsy*	Group 3 – 17.6
Gypsy-6_DSe	*D. sechellia*	*Ty3/Gypsy*	Group 2 – BLASTOPIA
Gypsy-7_DSe	*D. sechellia*	*Ty3/Gypsy*	Group 2 – MICROPIA

Gypsy-8_DSe	*D. sechellia*	*Ty3/Gypsy*	Group 3 – 17.6
Copia-1_DSim	*D. simulans*	*Ty1/Copia*	COPIA2_bis
Gypsy-1_DSim	*D. simulans*	*Ty3/Gypsy*	Group 3 – GYPSY
Gypsy-10_DSim	*D. simulans*	*Ty3/Gypsy*	Group 3 – GYPSY
Gypsy-11_DSim	*D. simulans*	*Ty3/Gypsy*	Group 3 – GYPSY
Gypsy-12_DSim	*D. simulans*	*Ty3/Gypsy*	Group 3 – 17.6
Gypsy-13_DSim	*D. simulans*	*Ty3/Gypsy*	Group 1 – OSVALDO
Gypsy-2_DSim	*D. simulans*	*Ty3/Gypsy*	Group 2 – MICROPIA
Gypsy-3_DSim	*D. simulans*	*Ty3/Gypsy*	Group 2 – SACCO
Gypsy-4_DSim	*D. simulans*	*Ty3/Gypsy*	Group 3 – 17.6
Gypsy-5_DSim	*D. simulans*	*Ty3/Gypsy*	Group 3 – 17.6
Gypsy-6_DSim	*D. simulans*	*Ty3/Gypsy*	Group 3 – 17.6
Gypsy-7_DSim	*D. simulans*	*Ty3/Gypsy*	? (too short)
Gypsy-8_DSim	*D. simulans*	*Ty3/Gypsy*	Group 3 – 412/MDG1
Gypsy-9_DSim	*D. simulans*	*Ty3/Gypsy*	? (no pol)
NINJA_DSim	*D. simulans*	*BEL/Pao*	NINJA
BEL-1_DTa	*D. takahashi*	*BEL/Pao*	NINJA
BEL-10_DTa	*D. takahashi*	*BEL/Pao*	BELMONDO2
BEL-11_DTa	*D. takahashi*	*BEL/Pao*	NINJA
BEL-12_DTa	*D. takahashi*	*BEL/Pao*	BELMONDO2
BEL-13_DTa	*D. takahashi*	*BEL/Pao*	NINJA
BEL-14_DTa	*D. takahashi*	*BEL/Pao*	BELMONDO2
BEL-15_DTa	*D. takahashi*	*BEL/Pao*	BELMONDO
BEL-16_DTa	*D. takahashi*	*BEL/Pao*	BEL
BEL-17_DTa	*D. takahashi*	*BEL/Pao*	NINJA
BEL-18_DTa	*D. takahashi*	*BEL/Pao*	NINJA
BEL-19_DTa	*D. takahashi*	*BEL/Pao*	BELMONDO
BEL-2_DTa	*D. takahashi*	*BEL/Pao*	NINJA
BEL-20_DTa	*D. takahashi*	*BEL/Pao*	BELMONDO2
BEL-21_DTa	*D. takahashi*	*BEL/Pao*	ROO/ROOA
BEL-22_DTa	*D. takahashi*	*BEL/Pao*	BATUMI / MAX
BEL-3_DTa	*D. takahashi*	*BEL/Pao*	BELMONDO2
BEL-4_DTa	*D. takahashi*	*BEL/Pao*	BELMONDO
BEL-5_DTa	*D. takahashi*	*BEL/Pao*	BELMONDO2
BEL-6_DTa	*D. takahashi*	*BEL/Pao*	NINJA
BEL-7_DTa	*D. takahashi*	*BEL/Pao*	DIVER2
BEL-8_DTa	*D. takahashi*	*BEL/Pao*	BELMONDO2
BEL-9_DTa	*D. takahashi*	*BEL/Pao*	BELMONDO2
Copia-1_DTa	*D. takahashi*	*Ty1/Copia*	COPIABIS
Copia-2_DTa	*D. takahashi*	*Ty1/Copia*	1731
Gypsy-1_DTa	*D. takahashii*	*Ty3/Gypsy*	Group 2 – MDG3
Gypsy-10_DTa	*D. takahashii*	*Ty3/Gypsy*	Group 1 – OSIRIS

Gypsy-11_DTa	*D. takahashii*	*Ty3/Gypsy*	Group 3 – 17.6
Gypsy-12_DTa	*D. takahashii*	*Ty3/Gypsy*	Group 3 – 17.6
Gypsy-13_DTa	*D. takahashii*	*Ty3/Gypsy*	Group 2 – MICROPIA
Gypsy-14_DTa	*D. takahashii*	*Ty3/Gypsy*	Group 2 – MICROPIA
Gypsy-15_DTa	*D. takahashii*	*Ty3/Gypsy*	Group 1 – ULYSSES
Gypsy-16_DTa	*D. takahashii*	*Ty3/Gypsy*	Group 2 – BICA
Gypsy-17_DTa	*D. takahashii*	*Ty3/Gypsy*	Group 2 – BLASTOPIA
Gypsy-18_DTa	*D. takahashii*	*Ty3/Gypsy*	Group 1 – OSVALDO
Gypsy-19_DTa	*D. takahashii*	*Ty3/Gypsy*	Group 1 – OSVALDO
Gypsy-2_DTa	*D. takahashii*	*Ty3/Gypsy*	Group 2 – MDG3
Gypsy-20_DTa	*D. takahashii*	*Ty3/Gypsy*	Group 1 – OSVALDO
Gypsy-3_DTa	*D. takahashii*	*Ty3/Gypsy*	Group 2 – BLASTOPIA
Gypsy-4_DTa	*D. takahashii*	*Ty3/Gypsy*	Group 3 – CHIMPO
Gypsy-5_DTa	*D. takahashii*	*Ty3/Gypsy*	Group 1 – OSIRIS
Gypsy-6_DTa	*D. takahashii*	*Ty3/Gypsy*	Group 1 – OSIRIS
Gypsy-7_DTa	*D. takahashii*	*Ty3/Gypsy*	Group 1 – OSIRIS
Gypsy-8_DTa	*D. takahashii*	*Ty3/Gypsy*	Group 2 – SACCO
Gypsy-9_DTa	*D. takahashii*	*Ty3/Gypsy*	Group 2 – MDG3
BEL-1_DVir	*D. virilis*	*BEL/Pao*	NINJA
BEL-2_DVir	*D. virilis*	*BEL/Pao*	DIVER2
BEL-3_DVir	*D. virilis*	*BEL/Pao*	DIVER2
BEL-4_DVir	*D. virilis*	*BEL/Pao*	NINJA
BEL-5_DVir	*D. virilis*	*BEL/Pao*	DIVER2
BEL1_DVir	*D. virilis*	*BEL/Pao*	DIVER2
Copia-1_Dvir	*D. virilis*	*Ty1/Copia*	Xanthias
Gypsy-1_DVir	*D. virilis*	*Ty3/Gypsy*	Group 3 – 412/MDG1
Gypsy-10_DVir	*D. virilis*	*Ty3/Gypsy*	Group 1 – ISIS-like
Gypsy-11_DVir	*D. virilis*	*Ty3/Gypsy*	Group 1 – ISIS
Gypsy-12_DVir	*D. virilis*	*Ty3/Gypsy*	Group 3 – GYPSY
Gypsy-13_DVir	*D. virilis*	*Ty3/Gypsy*	Group 2 – BICA
Gypsy-2_DVir	*D. virilis*	*Ty3/Gypsy*	Group 1 – ISIS-like
Gypsy-3_DVir	*D. virilis*	*Ty3/Gypsy*	Group 3 – GYPSY
Gypsy-4_DVir	*D. virilis*	*Ty3/Gypsy*	Group 2 – BLASTOPIA
Gypsy-5_DVir	*D. virilis*	*Ty3/Gypsy*	Group 3 – 17.6
Gypsy-6_DVir	*D. virilis*	*Ty3/Gypsy*	Group 2 – BLASTOPIA
Gypsy-7_DVir	*D. virilis*	*Ty3/Gypsy*	Group 3 – 412/MDG1
Gypsy-8_DVir	*D. virilis*	*Ty3/Gypsy*	Group 2 – MICROPIA
Gypsy-9_DVir	*D. virilis*	*Ty3/Gypsy*	Group 2 – MICROPIA
Gypsy1_DV	*D. virilis*	*Ty3/Gypsy*	Group 2 – MDG3
Gypsy2_DV	*D. virilis*	*Ty3/Gypsy*	Group 2 – MICROPIA
Gypsy3_DV	*D. virilis*	*Ty3/Gypsy*	Group 1 – OSVALDO
TV1_Dvir	*D. virilis*	*Ty3/Gypsy*	Group 3 – 17.6

ULYSSES_Dvir	*D. virilis*	*Ty3/Gypsy*	Group 1 – ULYSSES
BEL-1_DWil	*D. willistoni*	*BEL/Pao*	BATUMI / MAX
BEL-10_DWil	*D. willistoni*	*BEL/Pao*	BATUMI / MAX
BEL-11_DWil	*D. willistoni*	*BEL/Pao*	NINJA
BEL-12_DWil	*D. willistoni*	*BEL/Pao*	NINJA
BEL-13_DWil	*D. willistoni*	*BEL/Pao*	BEL
BEL-14_DWil	*D. willistoni*	*BEL/Pao*	BEL
BEL-15_DWil	*D. willistoni*	*BEL/Pao*	NINJA
BEL-16_DWil	*D. willistoni*	*BEL/Pao*	BEL
BEL-18_DWil	*D. willistoni*	*BEL/Pao*	BELMONDO2
BEL-19_DWil	*D. willistoni*	*BEL/Pao*	NINJA
BEL-2_DWil	*D. willistoni*	*BEL/Pao*	NINJA
BEL-20_DWil	*D. willistoni*	*BEL/Pao*	NINJA
BEL-21_DWil	*D. willistoni*	*BEL/Pao*	BATUMI / MAX
BEL-3_DWil	*D. willistoni*	*BEL/Pao*	BEL
BEL-4_DWil	*D. willistoni*	*BEL/Pao*	DIVER
BEL-5_DWil	*D. willistoni*	*BEL/Pao*	NINJA
BEL-6_DWil	*D. willistoni*	*BEL/Pao*	ROO/ROOA
BEL-7_DWil	*D. willistoni*	*BEL/Pao*	NINJA
BEL-8_DWil	*D. willistoni*	*BEL/Pao*	NINJA
BEL-9_DWil	*D. willistoni*	*BEL/Pao*	BELMONDO2
Copia-1_DWil	*D. willistoni*	*Ty1/Copia*	1731
Copia-2_DWil	*D. willistoni*	*Ty1/Copia*	1731
Copia-3_DWil	*D. willistoni*	*Ty1/Copia*	1731
Copia-4_DWil	*D. willistoni*	*Ty1/Copia*	COPIA2/COPIA2_bis
Copia-5_DWil	*D. willistoni*	*Ty1/Copia*	COPIA
Copia-6_DWil	*D. willistoni*	*Ty1/Copia*	COPIABIS
Copia-7_DWil	*D. willistoni*	*Ty1/Copia*	outsider
Copia-8_DWil	*D. willistoni*	*Ty1/Copia*	COPIA
Copia-9_DWil	*D. willistoni*	*Ty1/Copia*	COPIABIS
Gypsy-1_DWil	*D. willistoni*	*Ty3/Gypsy*	Group 1 – OSVALDO
Gypsy-10_DWil	*D. willistoni*	*Ty3/Gypsy*	Group 2 – BLASTOPIA
Gypsy-11_DWil	*D. willistoni*	*Ty3/Gypsy*	Group 2 – BICA
Gypsy-12_DWil	*D. willistoni*	*Ty3/Gypsy*	Group 2 – MDG3
Gypsy-13_DWil	*D. willistoni*	*Ty3/Gypsy*	Group 1
Gypsy-14_DWil	*D. willistoni*	*Ty3/Gypsy*	Group 3 – GYPSY
Gypsy-14B_DWil	*D. willistoni*	*Ty3/Gypsy*	? (no pol)
Gypsy-15_DWil	*D. willistoni*	*Ty3/Gypsy*	Group 3 – GYPSY
Gypsy-16_DWil	*D. willistoni*	*Ty3/Gypsy*	Group 2 – MDG3
Gypsy-17_DWil	*D. willistoni*	*Ty3/Gypsy*	Group 2 – MDG3
Gypsy-18_DWil	*D. willistoni*	*Ty3/Gypsy*	Group 1 – ISIS
Gypsy-19_DWil	*D. willistoni*	*Ty3/Gypsy*	Group 2 – BLASTOPIA

Gypsy-2_DWil	*D. willistoni*	*Ty3/Gypsy*	Group 1 – OSIRIS
Gypsy-20_DWil	*D. willistoni*	*Ty3/Gypsy*	Group 3 – GYPSY
Gypsy-21_DWil	*D. willistoni*	*Ty3/Gypsy*	Group 2 – BLASTOPIA
Gypsy-22_DWil	*D. willistoni*	*Ty3/Gypsy*	Group 3 – 17.6
Gypsy-23_DWil	*D. willistoni*	*Ty3/Gypsy*	Group 3 – 412/MDG1
Gypsy-24_DWil	*D. willistoni*	*Ty3/Gypsy*	Group 2 – SACCO
Gypsy-25_DWil	*D. willistoni*	*Ty3/Gypsy*	Group 2 – BLASTOPIA
Gypsy-26_DWil	*D. willistoni*	*Ty3/Gypsy*	Group 2 – SACCO
Gypsy-27_DWil	*D. willistoni*	*Ty3/Gypsy*	Group 3 – GYPSY
Gypsy-28_DWil	*D. willistoni*	*Ty3/Gypsy*	Group 2 – BLASTOPIA
Gypsy-29_DWil	*D. willistoni*	*Ty3/Gypsy*	Group 3 – 17.6
Gypsy-3_DWil	*D. willistoni*	*Ty3/Gypsy*	Group 2 – BLASTOPIA
Gypsy-30_DWil	*D. willistoni*	*Ty3/Gypsy*	Group 2 – MICROPIA
Gypsy-31_DWil	*D. willistoni*	*Ty3/Gypsy*	Group 2 – SACCO
Gypsy-32_DWil	*D. willistoni*	*Ty3/Gypsy*	Group 2 – BLASTOPIA
Gypsy-33_DWil	*D. willistoni*	*Ty3/Gypsy*	Group 2 – BLASTOPIA
Gypsy-34_DWil	*D. willistoni*	*Ty3/Gypsy*	Group 1 – ISIS
Gypsy-35_DWil	*D. willistoni*	*Ty3/Gypsy*	Group 1 – ULYSSES
Gypsy-36_DWil	*D. willistoni*	*Ty3/Gypsy*	Group 2 – MDG3
Gypsy-37_DWil	*D. willistoni*	*Ty3/Gypsy*	Group 1 – OSIRIS
Gypsy-38_DWil	*D. willistoni*	*Ty3/Gypsy*	Group 2 – BLASTOPIA
Gypsy-4_DWil	*D. willistoni*	*Ty3/Gypsy*	Group 2 – BLASTOPIA
Gypsy-42_DWil	*D. willistoni*	*Ty3/Gypsy*	Group 2 – BLASTOPIA
Gypsy-43_DWil	*D. willistoni*	*Ty3/Gypsy*	Group 2 – SACCO
Gypsy-44_DWil	*D. willistoni*	*Ty3/Gypsy*	Group 3 – GYPSY
Gypsy-45_DWil	*D. willistoni*	*Ty3/Gypsy*	Group 2 – SACCO
Gypsy-46_DWil	*D. willistoni*	*Ty3/Gypsy*	Group 2 – MICROPIA
Gypsy-47_DWil	*D. willistoni*	*Ty3/Gypsy*	Group 1 – OSIRIS
Gypsy-48_DWil	*D. willistoni*	*Ty3/Gypsy*	Group 1 – ULYSSES
Gypsy-49_DWil	*D. willistoni*	*Ty3/Gypsy*	Group 2 – MICROPIA
Gypsy-5_DWil	*D. willistoni*	*Ty3/Gypsy*	Group 3 – 412/MDG1
Gypsy-50_DWil	*D. willistoni*	*Ty3/Gypsy*	Group 3 – 412/MDG1
Gypsy-51_DWil	*D. willistoni*	*Ty3/Gypsy*	Group 2 – BLASTOPIA
Gypsy-52_DWil	*D. willistoni*	*Ty3/Gypsy*	Group 1 – ISIS
Gypsy-53_DWil	*D. willistoni*	*Ty3/Gypsy*	Group 2 – MDG3
Gypsy-54_DWil	*D. willistoni*	*Ty3/Gypsy*	Group 1 – ISIS-like
Gypsy-55_DWil	*D. willistoni*	*Ty3/Gypsy*	Group 2 – BLASTOPIA
Gypsy-56_DWil	*D. willistoni*	*Ty3/Gypsy*	Group 2 – BICA
Gypsy-57_DWil	*D. willistoni*	*Ty3/Gypsy*	Group 3 – 412/MDG1
Gypsy-58_DWil	*D. willistoni*	*Ty3/Gypsy*	Group 2 – BLASTOPIA
Gypsy-59_DWil	*D. willistoni*	*Ty3/Gypsy*	Group 2 – MDG3
Gypsy-6_DWil	*D. willistoni*	*Ty3/Gypsy*	Group 2 – MDG3

Gypsy-60_DWil	*D. willistoni*	*Ty3/Gypsy*	Group 3 – 17.6
Gypsy-61_DWil	*D. willistoni*	*Ty3/Gypsy*	Group 1 – OSVALDO
Gypsy-62_DWil	*D. willistoni*	*Ty3/Gypsy*	Group 3 – 412/MDG1
Gypsy-63_DWil	*D. willistoni*	*Ty3/Gypsy*	Group 3 – GYPSY
Gypsy-64_DWil	*D. willistoni*	*Ty3/Gypsy*	Group 2 – BLASTOPIA
Gypsy-7_DWil	*D. willistoni*	*Ty3/Gypsy*	Group 2 – BLASTOPIA
Gypsy-8_DWil	*D. willistoni*	*Ty3/Gypsy*	Group 2 – BLASTOPIA
Gypsy-9_DWil	*D. willistoni*	*Ty3/Gypsy*	Group 2 – SACCO
BEL-3_DYa	*D. yakuba*	*BEL/Pao*	BELMONDO2
BEL-4_DYa	*D. yakuba*	*BEL/Pao*	BELMONDO2
BEL-5_DYa	*D. yakuba*	*BEL/Pao*	BELMONDO2
BEL-6_DYa	*D. yakuba*	*BEL/Pao*	DIVER2
BEL1_Dya	*D. yakuba*	*BEL/Pao*	BATUMI / MAX
BEL2_DYa	*D. yakuba*	*BEL/Pao*	BATUMI / MAX
Copia-1_Dya = New_Xanthias_Dya	*D. yakuba*	*Ty1/Copia*	new Xanthias
Copia-1-DY	*D. yakuba*	*Ty1/Copia*	1731
Copia-2_DYa	*D. yakuba*	*Ty1/Copia*	COPIA
Gypsy_6B_Dya	*D. yakuba*	*Ty3/Gypsy*	Group 3 – GYPSY
Gypsy-21_DY	*D. yakuba*	*Ty3/Gypsy*	Group 1 – OSVALDO
Gypsy-21_DYa	*D. yakuba*	*Ty3/Gypsy*	Group 2 – MDG3
Gypsy-22_DY	*D. yakuba*	*Ty3/Gypsy*	Group 1 – OSIRIS
Gypsy-22_DYa	*D. yakuba*	*Ty3/Gypsy*	Group 2 – MICROPIA
Gypsy-23_DY	*D. yakuba*	*Ty3/Gypsy*	Group 1 – OSIRIS
Gypsy-23_DYa	*D. yakuba*	*Ty3/Gypsy*	Group 2 – MICROPIA
Gypsy-24_DY	*D. yakuba*	*Ty3/Gypsy*	Group 3 – GYPSY
Gypsy-24_DYa	*D. yakuba*	*Ty3/Gypsy*	Group 2 – SACCO
Gypsy-25_DYa	*D. yakuba*	*Ty3/Gypsy*	Group 2 – MDG3
Gypsy-26_DYa	*D. yakuba*	*Ty3/Gypsy*	Group 1 – OSVALDO
Gypsy-27_DYa	*D. yakuba*	*Ty3/Gypsy*	Group 2 – MDG3
Gypsy-28_DYa	*D. yakuba*	*Ty3/Gypsy*	Group 3 – 17.6
Gypsy-29_DYa	*D. yakuba*	*Ty3/Gypsy*	Group 2 – BICA
Gypsy-30_DYa	*D. yakuba*	*Ty3/Gypsy*	Group 1 – ULYSSES
Gypsy-31_DYa	*D. yakuba*	*Ty3/Gypsy*	Group 3 – 17.6
Gypsy-32_DYa	*D. yakuba*	*Ty3/Gypsy*	Group 1 – OSIRIS
Gypsy1_Dya	*D. yakuba*	*Ty3/Gypsy*	Group 2 – BLASTOPIA
Gypsy10_Dya	*D. yakuba*	*Ty3/Gypsy*	Group 1 – OSIRIS
Gypsy11_Dya	*D. yakuba*	*Ty3/Gypsy*	Group 3 – GYPSY
Gypsy12_Dya	*D. yakuba*	*Ty3/Gypsy*	Group 2 – BLASTOPIA
Gypsy13_Dya	*D. yakuba*	*Ty3/Gypsy*	Group 1 – ISIS
Gypsy14_Dya	*D. yakuba*	*Ty3/Gypsy*	Group 2 – BLASTOPIA
Gypsy15_Dya	*D. yakuba*	*Ty3/Gypsy*	Group 2 – MDG3
Gypsy16_Dya	*D. yakuba*	*Ty3/Gypsy*	Group 3 – GYPSY

Gypsy17_Dya	*D. yakuba*	*Ty3/Gypsy*	Group 2 – SACCO
Gypsy18_Dya	*D. yakuba*	*Ty3/Gypsy*	Group 1 – OSIRIS
Gypsy19_Dya	*D. yakuba*	*Ty3/Gypsy*	Group 2 – MDG3
Gypsy2_Dya	*D. yakuba*	*Ty3/Gypsy*	Group 2 – BLASTOPIA
Gypsy20_Dya	*D. yakuba*	*Ty3/Gypsy*	Group 3 – 17.6
Gypsy3_Dya	*D. yakuba*	*Ty3/Gypsy*	Group 3
Gypsy4_Dya	*D. yakuba*	*Ty3/Gypsy*	Group 1 – OSVALDO
Gypsy5_Dya	*D. yakuba*	*Ty3/Gypsy*	Group 1 – ISIS-like
Gypsy6_Dya	*D. yakuba*	*Ty3/Gypsy*	Group 1 – ISIS
Gypsy7_Dya	*D. yakuba*	*Ty3/Gypsy*	Group 2 – SACCO
Gypsy8_Dya	*D. yakuba*	*Ty3/Gypsy*	Group 3 – 17.6
Gypsy9_Dya	*D. yakuba*	*Ty3/Gypsy*	Group 2 – MICROPIA
Pifo_Dyak	*D. yakuba*	*Ty3/Gypsy*	Group 3 – 17.6
