# Supplementary material for: Evolutionary history of LTR-retrotransposons among 20 Drosophila species
Source: Mob DNA. 2017 Apr 27;8:7. doi: 10.1186/s13100-017-0090-3 (PMC5408442; doi:10.1186/s13100-017-0090-3)
Supplement: Supplementary file 3 — Graphical matrix view generated by the VHICA method for HT cases in the A) Ty1/Copia superfamily, B) BEL/Pao and C,D,E) Ty3/Gypsy superfamilies. (PDF 779 kb) [file 13100_2017_90_MOESM3_ESM.pdf]

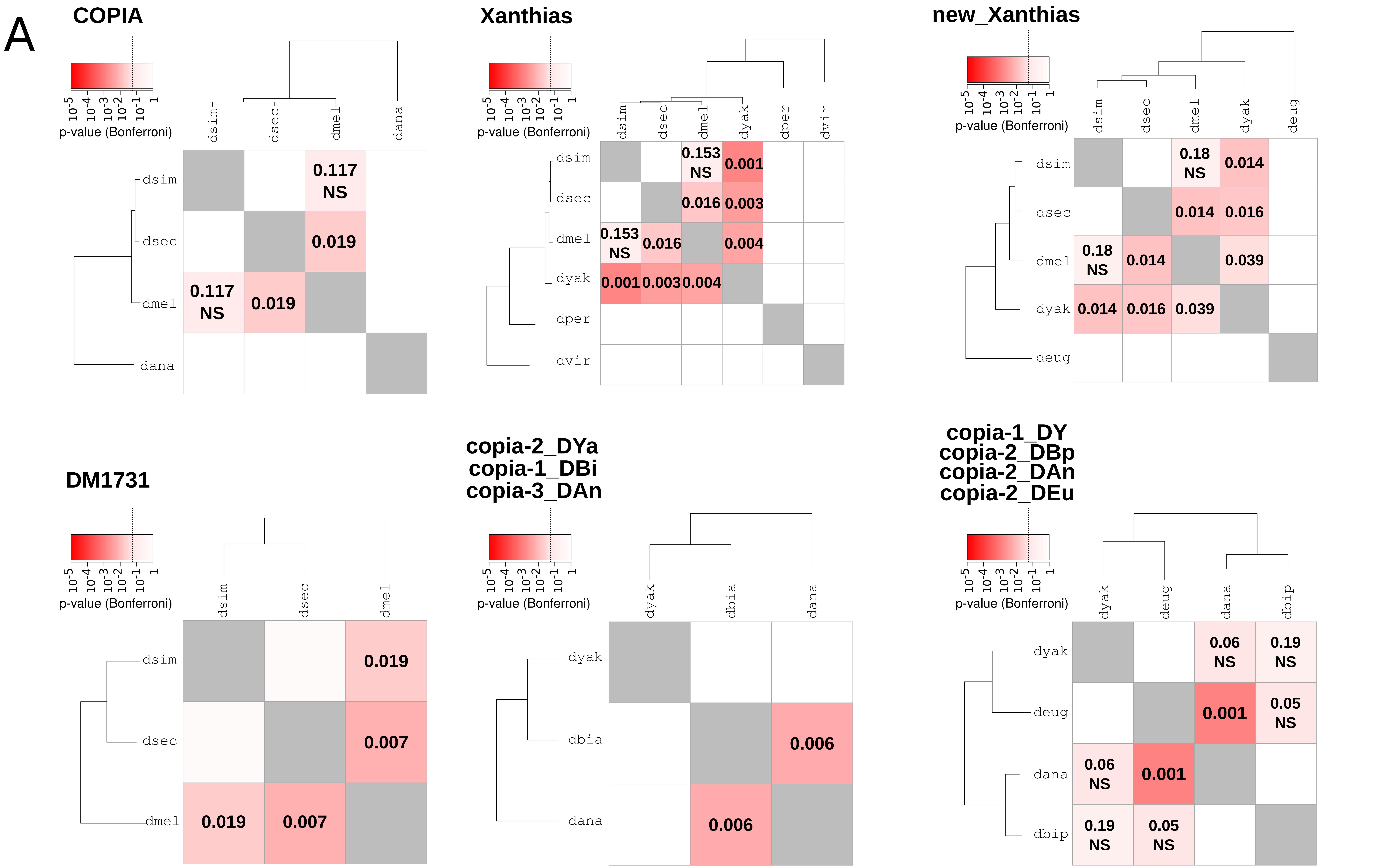

Figure S1: graphical matrix view generated by the VHICA method for HT cases in the A) *Ty1/Copia* superfamily, B) *BEL/Pao* and C,D,E) *Ty3/Gypsy* superfamilies.

The matrix square represent all pairwise comparisons. Each square is colored according to the P-value calculated for the null hypothesis of vertical transmission. Red color indicates cases of potential horizontal transfers. When the square is divided into rectangles, each one represents one sublineage (meaning sequence variant of a given family present in the given species). The dot line indicates threshold (P_value = 0.05) to discriminate between vertical transmission and HT. We have indicated in the red squares the computed P_values, indicating those that are non significant (NS). For more details see Wallau et al. 2016

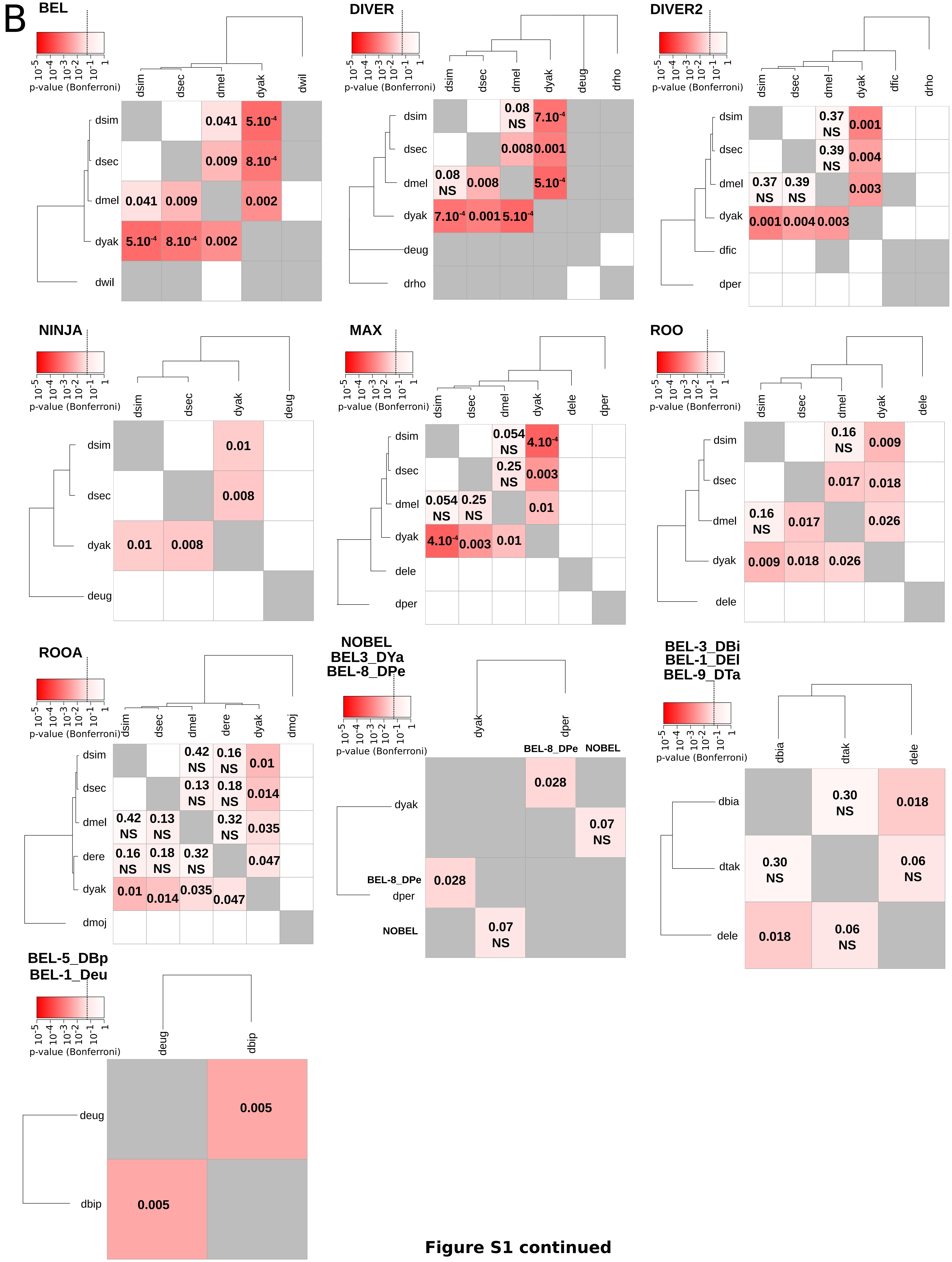

Figure S1 continued

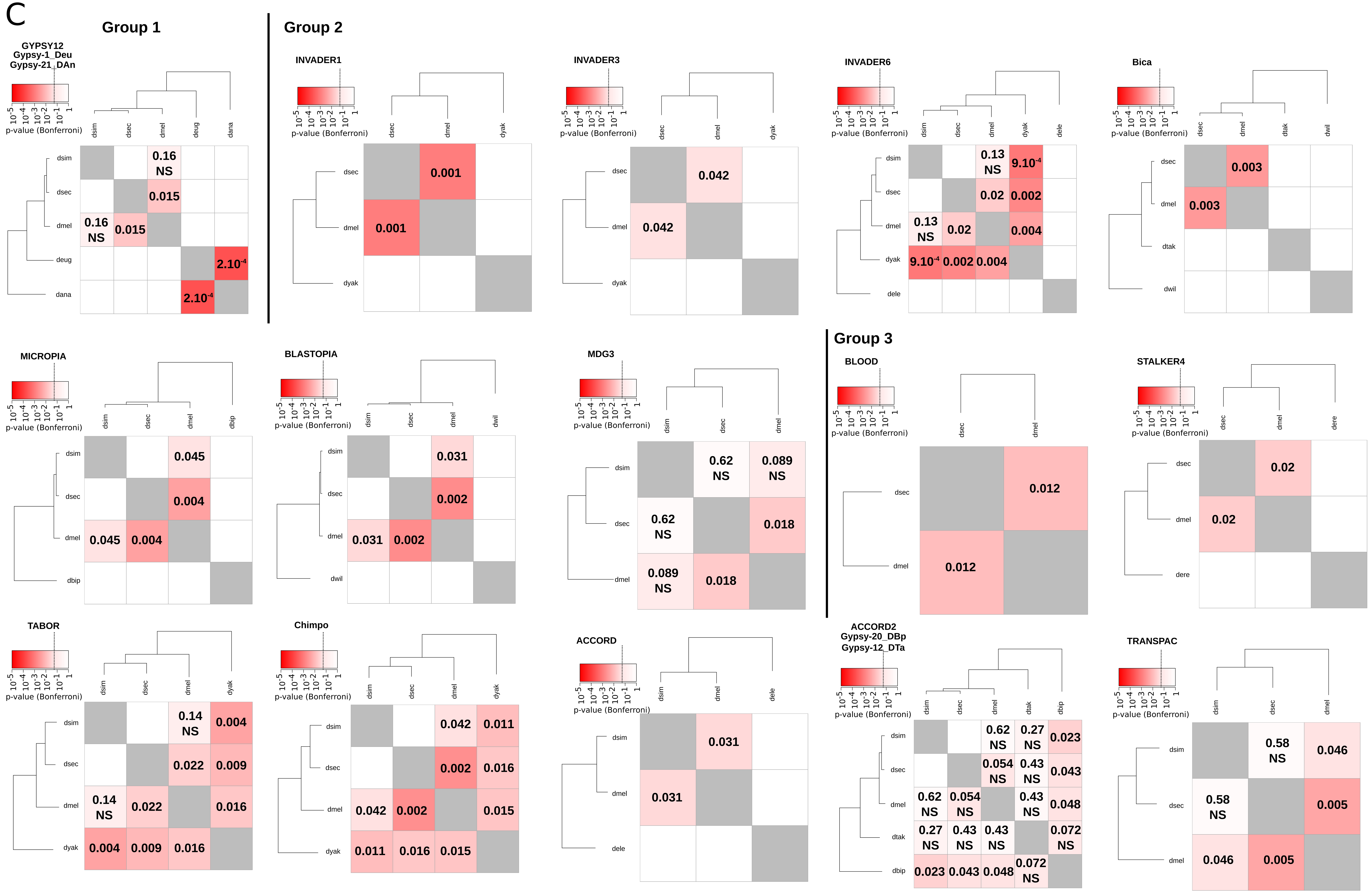

Figure S1 continued

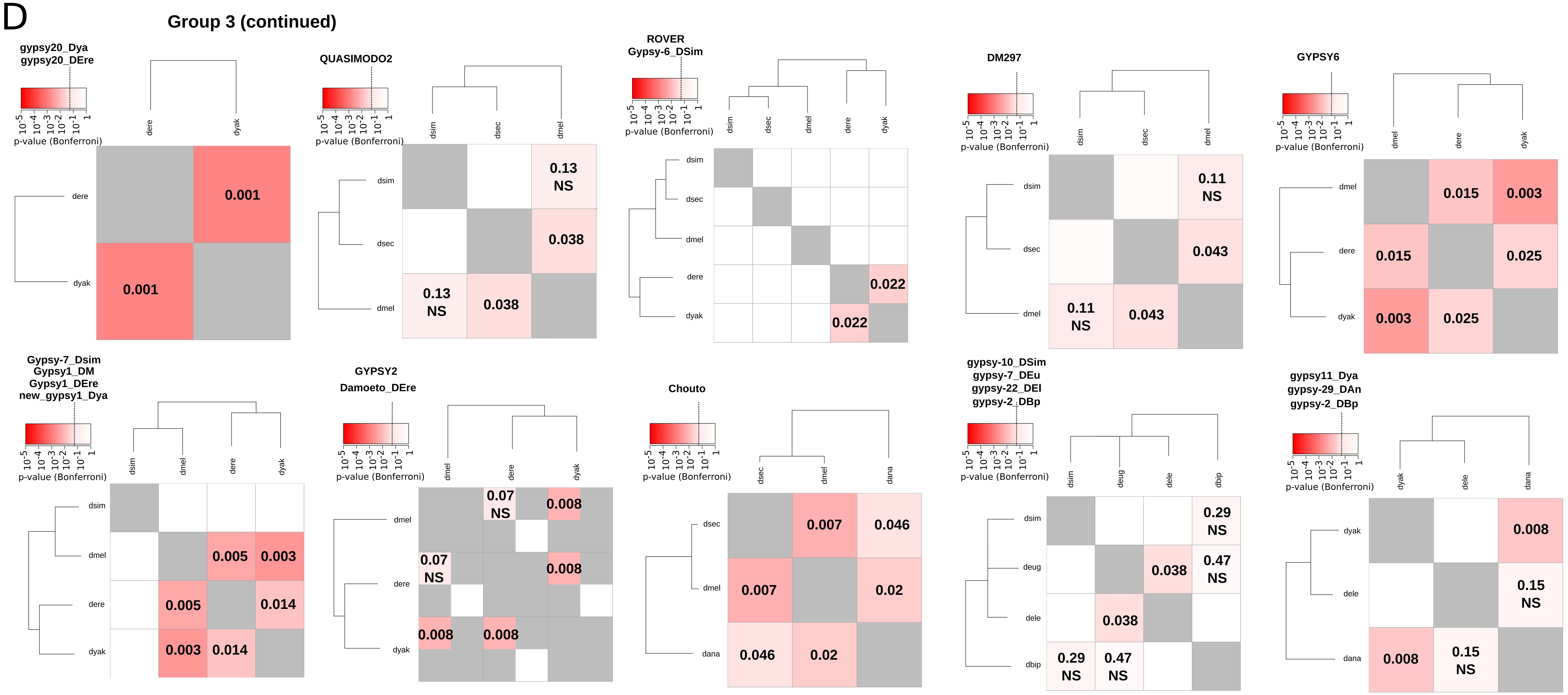

Figure S1 continued

E

Group 3 (continued)

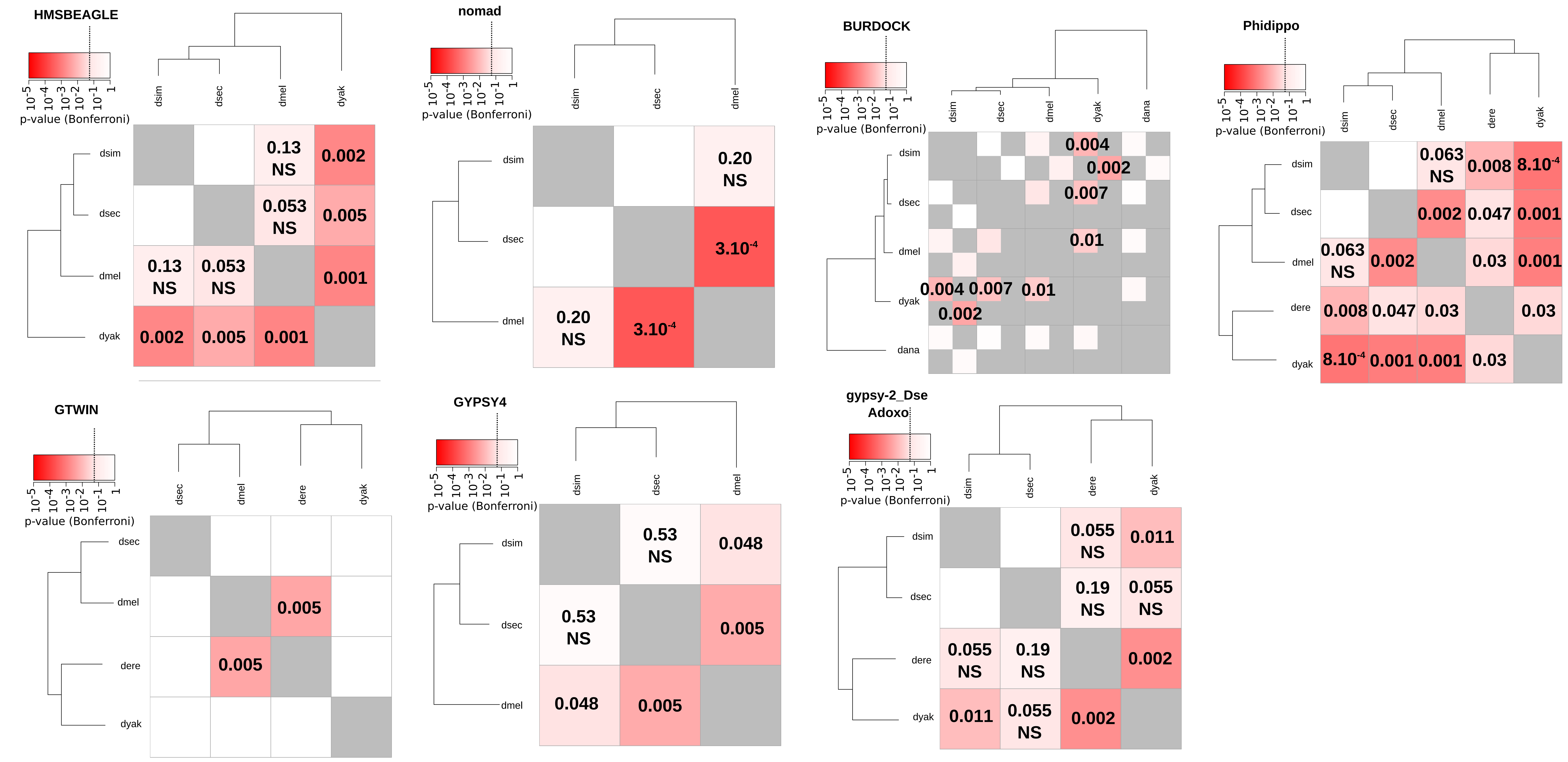

Figure S1 continued
