# Supplementary material for: Evolutionary history of LTR-retrotransposons among 20 Drosophila species
Source: Mob DNA. 2017 Apr 27;8:7. doi: 10.1186/s13100-017-0090-3 (PMC5408442; doi:10.1186/s13100-017-0090-3)

A) Ty1/Copia

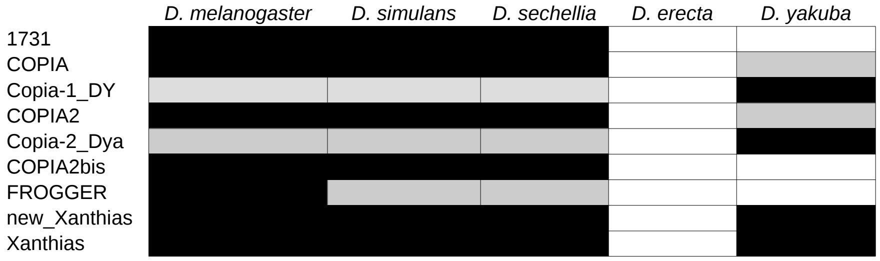

Supplementary figure S2: pattern of presence (black), absence (white) or traces (gray) of a given TE in the species of the *melanogaster* subgroup for A) Ty1/Copia, B) BEL/Pao, and C) Ty3/Gypsy superfamilies

B) BEL/Pao

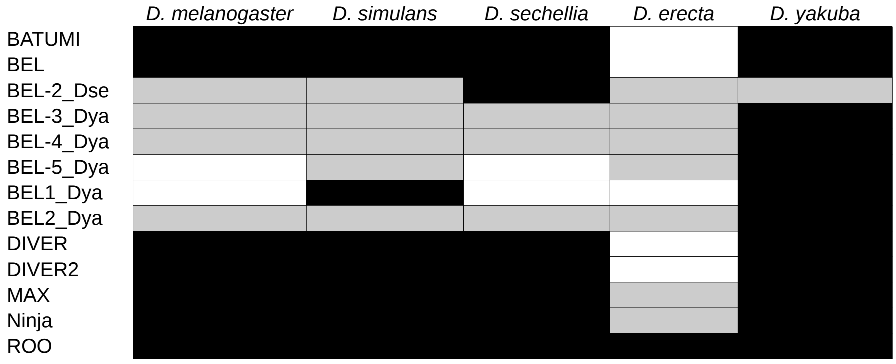
D. melanogaster
D. simulans
D. sechellia
D. erecta
D. yakuba
BATUMI
BEL
BEL-2_Dse
BEL-3_Dya
BEL-4_Dya
BEL-5_Dya
BEL1_Dya
BEL2_Dya
DIVER
DIVER2
MAX
Ninja
ROO

C) Ty3/Gypsy

group 1 "OSVALDO/ULYSSES"

		D. melanogaster	*D. simulans*	*D. sechellia*	*D. erecta*	*D. yakuba*
ISIS	Gypsy13_Dya					
ISIS	Gypsy6_Dya					
ISIS-like	Gypsy31_Dya					
ISIS-like	Gypsy5_Dya					
ISIS-like	Isislike_Dya					
OSIRIS	Gypsy-22_DY					
OSIRIS	Gypsy-23_DY					
OSIRIS	Gypsy-32_Dya					
OSIRIS	Gypsy10_Dya					
OSIRIS	GYPSY12					
OSIRIS	Gypsy18_Dya					
OSVALDO	Gypsy-21_DY					
OSVALDO	Gypsy-26_Dya					
OSVALDO	Gypsy4_Dya					
ULYSSES	Gypsy-30_Dya					

group 2 "MICROPIA/SACCO"

		D. melanogaster	*D. simulans*	*D. sechellia*	*D. erecta*	*D. yakuba*
BICA	Bica					
BICA	Gypsy-29_Dya					
BLASTOPIA	BLASTOPIA					
BLASTOPIA	Gypsy1_Dya					
BLASTOPIA	Gypsy12_Dya					
BLASTOPIA	Gypsy14_Dya					
BLASTOPIA	Gypsy2_Dya					
BLASTOPIA	Gypsy-6_Dse					
MICROPIA	Gypsy-2_Dsim					
MICROPIA	Gypsy-22_Dya					
MICROPIA	Gypsy-23_Dya					
MICROPIA	Gypsy-4_Dse					
MICROPIA	Gypsy9_Dya					
MICROPIA	INVADER1					
MICROPIA	INVADER6					
MICROPIA	MICROPIA					
MDG3	Gypsy-21_Dya					
MDG3	Gypsy-25_Dya					
MDG3	Gypsy-27_Dya					
MDG3	Gypsy15_Dya					
MDG3	Gypsy19_Dya					
MDG3	INVADER2					
MDG3	INVADER3					
MDG3	MDG3					
SACCO	Gypsy7_Dya					
SACCO	Gypsy17_Dya					
SACCO	Sacco					
SACCO	Saccobis					

Group 3 errantiviridae/412

		D. melanogaster	*D. simulans*	*D. sechellia*	*D. erecta*	*D. yakuba*
17.6	17.6					
17.6	ACCORD					
17.6	ACCORD2					
17.6	DM297					
17.6	Gypsy-28_Dya					
17.6	Gypsy-31_Dya					
17.6	Gypsy-4_Dsim					
17.6	Gypsy-5_Dse					
17.6	GYPSY5					
17.6	Gypsy8_Dya					
17.6	Pifo					
17.6	QUASIMODO					
17.6	QUASIMODO2					
17.6	ROVER					
17.6	TirantC					

17.6	TRANSPAC
17.6	ZAM
?	Gypsy3_Dya
412/MDG1	blood
412/MDG1	Gypsy-8_Dsim
412/MDG1	MDG1
412/MDG1	STALKER2
412/MDG1	STALKER4
412/MDG1	TABOR
CHIMPO	Chimpo
GYPSY	BURDOCK
GYPSY	Chouto
GYPSY	GTWIN
GYPSY	GYPSY
GYPSY	gypsy-1_Dse
GYPSY	Gypsy-1_Dsim
GYPSY	Gypsy-10_Dsim
GYPSY	Gypsy-11_Dsim
GYPSY	Gypsy11_Dya
GYPSY	GYPSY2
GYPSY	GYPSY3
GYPSY	Gypsy-1A_DSe
GYPSY	GYPSY4
GYPSY	GYPSY6
GYPSY	HMSBEAGLE
GYPSY	nomad

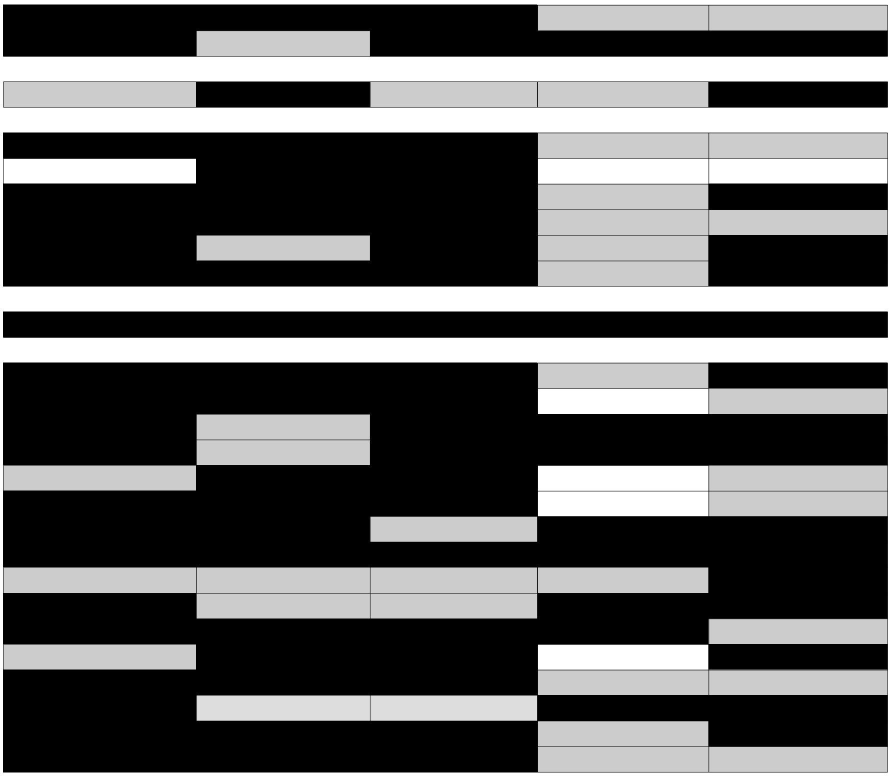

Supplement: Supplementary file 4 — Pattern of presence (black), absence (white) or traces (gray) of a given TE in the species of the melanogaster subgroup for A) Ty1/Copia, B) BEL/Pao, and C) Ty3/Gypsy superfamilies. (PDF 50 kb) [file 13100_2017_90_MOESM4_ESM.pdf]
