# Supplementary figures and images for: Evolutionary history of LTR-retrotransposons among 20 Drosophila species
Source: Mob DNA. 2017 Apr 27;8:7. doi: 10.1186/s13100-017-0090-3 (PMC5408442; doi:10.1186/s13100-017-0090-3)

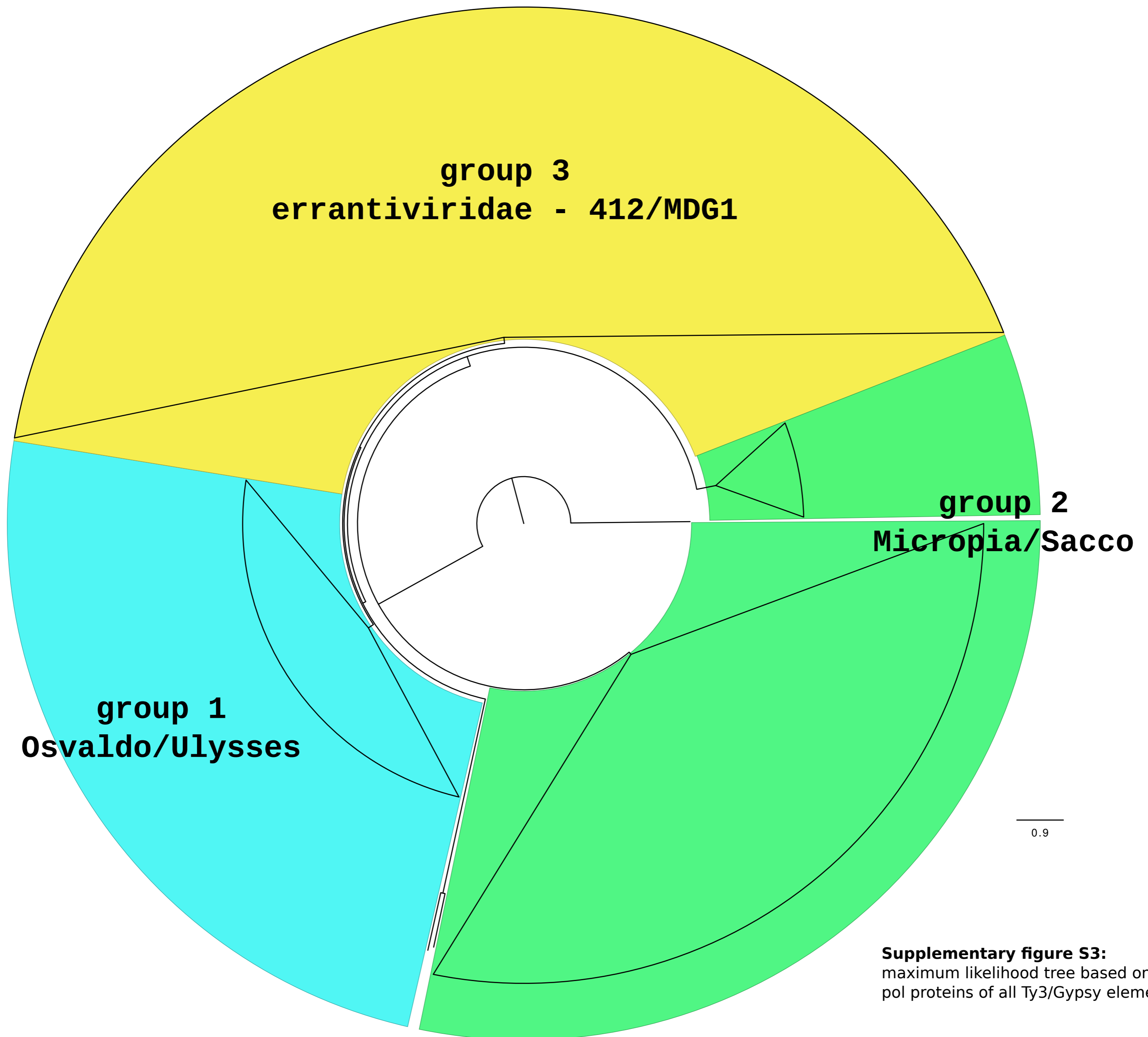

Supplement: Supplementary file 5 — Maximum likelihood treee based on the pot proteins of all Ty3/Gypsy elements. (PDF 35 kb) [file 13100_2017_90_MOESM5_ESM.pdf]
