# Supplementary material for: Evolutionary history of LTR-retrotransposons among 20 Drosophila species
Source: Mob DNA. 2017 Apr 27;8:7. doi: 10.1186/s13100-017-0090-3 (PMC5408442; doi:10.1186/s13100-017-0090-3)
Supplement: Supplementary file 6 — Single copy orthologous genes from the Drosophila genomes used in the dS estimate in the VHICA method. (PDF 231 kb) [file 13100_2017_90_MOESM6_ESM.pdf]

Supplementary Table S2: Single copy orthologous genes from the Drosophila genomes used in the dS estimate in the VHICA method.

Single Copy Orthologous Host Gene	Full name	Flybase Name
abd-A	abdominal A	FBgn0000014
Abl	Abl tyrosine kinase	FBgn0000017
nAChRβ1	nicotinic Acetylcholine Receptor β1	FBgn0000038
al	aristaless	FBgn0000061
bib	big brain	FBgn0000180
bur	burgundy	FBgn0000239
D	Dichaete	FBgn0000411
Ddc	dopa decarboxylase	FBgn0000422
dib	disembodied	FBgn0000449
E(z)	Enhancer of zeste	FBgn0000629
grau	grauzone	FBgn0001133
bap	bagpipe	FBgn0004862
DNApol-α73	DNA polymerase α 73kD	FBgn0005696
Nap1	Nucleosome assembly protein 1	FBgn0015268
CG5861	CG5861	FBgn0015338
RpS21	Ribosomal protein S21	FBgn0015521
AstA	Allatostatin A	FBgn0015591
Lis-1	Lissencephaly-1	FBgn0015754
EloB	Elongin B	FBgn0023212
Lim1	Lim1	FBgn0026411
Rab35	Rab35	FBgn0031090
CG9586	CG9586	FBgn0032101
Vps20	Vacuolar protein sorting 20	FBgn0034744
Art7	Arginine methyltransferase 7	FBgn0034817
Vdup1	Vitamin D3 up-regulated protein 1	FBgn0035103
pyx	pyrexia	FBgn0035113
cln3	cln3	FBgn0036756
Usp12-46	Ubiquitin-specific protease 12/46 ortholog	FBgn0039025
bc10	bc10	FBgn0040239
Gclc	Glutamate-cysteine ligase catalytic subunit	FBgn0040319
